# Evidence for Drop‐Like Nuclear Deformation in Sheared Endothelial Monolayers

**DOI:** 10.1002/smll.202506536

**Published:** 2025-12-26

**Authors:** Mohammad Mohajeri, Ting‐Ching Wang, Pooja Agarwal, Simran Kaur, Ankit Kumar, Richard B. Dickinson, Abhishek Jain, Tanmay P. Lele

**Affiliations:** ^1^ Department of Biomedical Engineering Texas A&M University College Station Texas USA; ^2^ Artie Mcferrin Department of Chemical Engineering Texas A&M University College Station Texas USA; ^3^ Department of Molecular and Cellular Biology University of California Berkeley California USA; ^4^ Department of Electrical Engineering & Computer Science University of California Berkeley California USA; ^5^ Department of Chemical Engineering University of Florida Gainesville Florida USA; ^6^ Department of Cardiovascular Sciences Houston Methodist Research Institute Houston Methodist Hospital Houston Texas USA; ^7^ Department of Medical Physiology Texas A&M Health Science Center Bryan Texas USA; ^8^ Department of Translational Medical Sciences Texas A&M University Houston Texas USA; ^9^ School of Engineering Medicine Texas A&M University Houston Texas USA

**Keywords:** endothelial cells, lamin A/C, mechanotransduction, nuclear mechanics, shear stress

## Abstract

Shear stress imparted by blood flow tends to smoothen endothelial monolayers, a response classically attributed to reduced nuclear height and nuclear reorientation along flow. However, the mechanical basis remains unclear. Here, we tested predictions of the nuclear drop model—which posits that nuclear shape changes occur at constant volume and surface area—in human umbilical vein endothelial cells (HUVECs) under physiological shear stress. HUVEC nuclear morphologies varied from smooth, flat nuclei to wrinkled, tall ones. Applying shear stress reduced the frequency of tall, wrinkled nuclei, explaining the population‐level decrease in nuclear height. Lamin A/C–depleted nuclei are highly irregular and failed to recover shapes postindentation on PDMS microposts, suggesting that lamin A/C confers nuclear surface tension. Nuclear volume and surface area remained constant under shear, consistent with the drop model, and a computational model based on these constraints successfully predicted observed nuclear shapes. Neither lamin A/C nor lamin B1 depletion prevented shear‐induced YAP nuclear localization; instead, shear detached poorly spread cells, increasing spreading, focal adhesion assembly, and cytoskeletal tension in the remaining cells, thereby promoting YAP nuclear localization. These findings revise classical interpretations of flow‐induced endothelial smoothing and show that flow‐induced YAP nuclear localization results from increased cell spreading rather than nuclear deformation.

## Graphical abstract

Physiological shear stress smoothens endothelial monolayers by selectively eliminating less‐spread cells with tall, wrinkled nuclei. Nuclear volume and surface area remain constant, consistent with drop‐like deformation governed by geometric constraints. Lamin A/C provides nuclear surface tension, while shear‐induced YAP nuclear localization arises indirectly from enhanced spreading, focal adhesion assembly, and cytoskeletal tension.

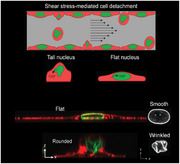

## 1 Introduction

Endothelial cells (ECs) are constantly exposed to fluid shear stress [1, 2]. As early as 1994, Barbee et al. reported that shear stress reduces the height of ECs and their nuclei [3]. However, whether this height reduction reflects direct vertical compression of nuclei, which are the tallest part in EC monolayers [3], remains unresolved. The shape of the nucleus in the x‐y plane is also altered such that elliptical nuclei align with flow [4]; this alignment has been proposed to minimize their hydrodynamic resistance [5]. Such changes in nuclear shape, which are a response of the nucleus to cellular mechanical forces [6, 7], are determined by the mechanical properties of the nucleus.

Contrary to typical mechanical models of the nucleus, which assume a smooth spherical starting shape that deforms into flattened morphologies [8, 9], we and others have reported that the rounded shape of the nucleus has wrinkles in it, in cell types including fibroblasts, cancer cells, and stem cells [10, 11, 12, 13, 14]. These wrinkles, which develop post‐mitosis during nuclear assembly, smoothen out as the cell spreads and flattens the nucleus. The unfolding of the wrinkles allows the nucleus to flatten without stretching the lamina. Nuclear shape changes occur at near constant surface area and volume, and are arrested once the lamina is fully unfolded [10, 11]. These features have motivated the formulation of the nuclear drop model, in which nuclear shape changes occur under the constraints of constant area and volume. A taut lamina is nearly inextensible owing to the presence of lamin A/C [10, 11, 15], and supports an in‐plane tension which is balanced by nuclear pressure. In this model, elastic resistance to shear deformation of chromatin and other structures in the nucleus is considered to be minimal over the time scales of nuclear shaping in cells [16].

Whether these features of the nuclear drop model apply to nuclear deformations in ECs under physiological levels of shear stress is not known. Here, we tested the predictions of this model in endothelial monolayers subjected to shear stress. Consistent with the drop model, the lamina wrinkled upon rounding of HUVECs through disruption of F‐actin networks, even as the nuclear volume and surface area both remained constant. The lamina was similarly wrinkled in rounded HUVECs in monolayers in a vessel‐on‐chip model. We found that the apparent nuclear flattening under flow previously reported can be attributed to a population‐level effect due to a selective detachment of less‐spread cells, which are more likely to contain wrinkled nuclei and tall nuclear profiles. These results offer a new explanation for Barbee et al.’s classical finding [3]: average nuclear height decreases under shear because poorly spread cells with wrinkled nuclei detach, and not because individual nuclei are compressed. Flow therefore causes an overall smoothing of the HUVEC monolayer.

To test whether lamin A/C confers the surface tension on the endothelial “nuclear drop,” we depleted it in HUVECs and analyzed nuclear shapes on PDMS micropost‐substrates [11, 15]. Lamin A/C‐depleted nuclei retained indentations after deformation around the microposts and failed to re‐adopt smooth contours, indicating a loss of surface tension consistent with the drop model. Although lamin A/C depleted nuclei aligned when shear flows were applied to cells, they no longer exhibited the characteristic smooth lamina of wild‐type cells.

Finally, we examined the consequences of these shape changes for EC mechanotransduction. Yes‐associated protein (YAP) translocated to the endothelial nucleus under shear application. We attribute this to a selection effect: shear stress eliminates poorly spread cells, which are more likely to exhibit wrinkled nuclei and weaker adhesion. This loss enables increased cell spreading in the remaining cells. These well‐spread cells assemble more focal adhesions, have increased myosin activation, and exhibit increased YAP nuclear localization [17]. Overall, YAP nuclear localization in this context reflects an indirect, population‐level consequence of cell spreading, rather than a direct result of nuclear deformation under force.

## 2 Results

### 2.1 Shear Stress Reduces the Incidence of Laminar Wrinkling in Endothelial Monolayers

**FIGURE 1:**
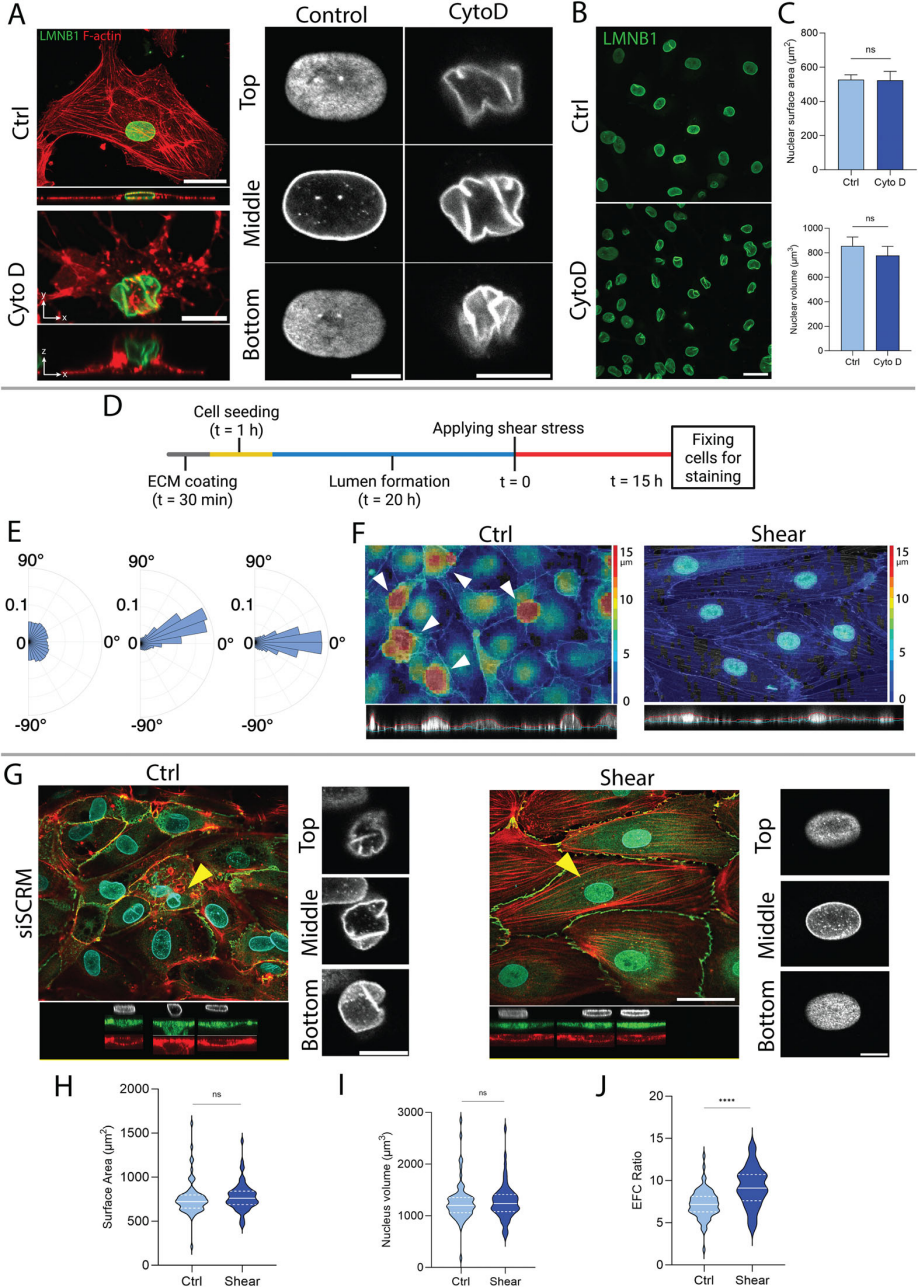
Endothelial monolayers exhibit reduced laminar wrinkling in response to shear stress. (A) x‐y and x‐z views of the control and cytochalasin D‐treated HUVECs stained for lamin B1 (green) and F‐actin (red). Top: control; scale bar is 30 µm. Bottom: cytochalasin D treated; scale bar is 10 µm. Closeup views demonstrate three confocal planes of a lamin B1‐stained nucleus showing taut and irregular contours in control and cytochalasin D‐treated EC, respectively. (B) Confocal images of lamin B1‐stained nuclei in control and cytochalasin D‐treated ECs. Scale bar is 30 µm. (C) Quantification of nuclear surface area and nuclear volume corresponding to the groups shown in (B). *n* = 14 and 23 for ctrl and cytoD, respectively, from three independent experiments. Error bars, SEM. *p*‐values 0.107 and 0.219, respectively, by Mann–Whitney *U‐*test. (D) Diagram shows the experimental timeline for the application of shear stress to HUVECs. (E) Radar plots show F‐actin–based alignment of HUVECs under shear stresses of 0.1, and 1 dyne/cm^2^ (10 images were analyzed for each condition from three independent experiments). (F) Color‐coded height maps of the apical surface of HUVEC monolayers under static (ctrl) conditions and shear stress (1 dyne/cm^2^), quantified from 3D confocal microscopy z‐stack images. Arrows point to the taller nuclei in the monolayer. (G) Confocal images of stained lamin B1(cyan), F‐actin (red), and VE‐Cadherin (green) in HUVEC monolayers for two different conditions, left: control, right: shear stress. Scale bar is 40 µm. Close‐up views show three confocal planes of the nuclei indicated by yellow arrows. Scale bar is 10 µm. Violin plots showing of H) nuclear surface area, (I) volume, and (J) EFC ratio for HUVECs cultured in control (*n* = 69 cells) and shear stress (*n* = 65 cells) conditions, from three independent experiments. ns *p* > 0.05, ^****^ *p* < 0.0001 by Mann–Whitney U test.

To test whether the nuclear drop model applies to ECs, we first asked whether wrinkling of the nuclear lamina occurs during nuclear rounding—as predicted by the nuclear drop model [18]. Treatment of HUVECs with cytochalasin D to disrupt F‐actin and induce rapid rounding (in ∼20 min) revealed prominent laminar wrinkling in z‐sections, as visualized by LMNB1 staining (Figure 1A,B). In contrast, control cells with flattened nuclei exhibited a smooth lamina with near constant mean curvatures in the x‐y plane. Consistent with the law of Laplace, these shapes suggest the presence of nuclear surface tension in control HUVECs that is balanced by the pressure difference across the nuclear envelope [15, 18, 19]. Importantly, nuclear surface area and volume (quantified from confocal images; see methods) were unchanged between wrinkled and smooth nuclei (Figure 1C). These results are consistent with the prediction of the drop model that a) nuclear shape transformations occur at constant volume and area, and b) a rounding of the flat nucleus must necessarily induce laminar wrinkling [20].

We next examined whether similar relationships hold in endothelial monolayers exposed to flow. HUVECs were cultured in a vessel‐on‐chip system [21, 22] and subjected to laminar shear stress (Figure 1D). HUVECs were fixed and stained for F‐actin, and their alignment under shear stress was quantified (Figure S1A). Cells began to align with flow at shear stresses as low as 0.1 dyne/cm^2^, and were fully aligned with the flow at shear stresses of 1 dyne/cm^2^ (Figure 1E); the latter value is consistent with magnitudes of shear stresses experienced by venous ECs in vivo [23]. Figure 1F shows color‐coded height maps of the apical surface of HUVEC monolayers (quantified from 3D confocal microscopy of F‐actin‐labeled cells) under static (no shear) conditions and under shear stress of 1 dyne/cm^2^. Colors represent the distance from the bottom glass substrate to the top surface of the cells, with warmer hues (red/yellow) indicating greater height and cooler hues (blue/green) indicating flatter regions. In the absence of shear, the monolayer featured several tall cells that extended above the plane of the monolayer, while in the presence of shear, such tall cells were absent. The x–z cross‐section shown below the images confirmed the presence of roughness in the monolayer in static conditions and a clear smoothing of the monolayer under shear stress. Consistent with this visual smoothing, the standard deviation of cell height was significantly lower under shear compared with static monolayers (mean SD: 1.18 ± 0.12 um for shear vs. mean SD:1.93 ± 0.06 um for no shear; *p *= 0.02 by permutation test ). Together, these data confirm early observations [3] that shear stress smoothens endothelial monolayers.

Given that nuclei are generally the tallest part of the endothelial cell [3], we examined endothelial nuclear shape by immunofluorescence microscopy of lamin B1. While many cells in unsheared (control) monolayers also contained nuclei with a smooth lamina, some less spread cells featured vertically rounded nuclei with a wrinkled lamina (Figure 1G, Ctrl). Shear flows aligned nuclei in HUVECs under shear stress (Figure S1B). These nuclei had a smooth lamina (Figure 1G, shear) suggestive of laminar tension. Quantitative analysis showed that nuclear surface area and volume (Figure 1H,I) were unchanged between sheared and unsheared conditions.

To quantify the change in nuclear wrinkling, we segmented nuclear contours and performed an Elliptical Fourier analysis, which we have recently described [24, 25]. We calculated an Elliptical Fourier coefficient (EFC ratio) defined as the ratio of the sum of the major and minor axis of the first elliptical harmonic to the sum of the rest of the harmonics required to describe the closed irregular contour (the higher the EFC ratio, the smoother the contour). A mean EFC ratio was calculated per nucleus from quantifications of an EFC ratio for each of three different confocal planes of a given lamin‐stained nucleus. Shear stress significantly increased the mean elliptical Fourier coefficient (EFC) ratio of nuclei (Figure 1J; the EFC ratio is inversely proportional to contour irregularity), supporting a population‐level decrease in the proportion of wrinkled nuclei. Overall, these results suggest that a geometric rounding of the nucleus induces wrinkles in the nuclear lamina and that shear stress reduces the occurrence of poorly spread cells with wrinkled, rounded nuclei in HUVEC monolayers. This likely accounts for the observed smoothening of the monolayers under shear stress in Figure 1F.

### 2.2 Lamin A/C Is Required for Nuclear Surface Tension in HUVECs

**FIGURE 2:**
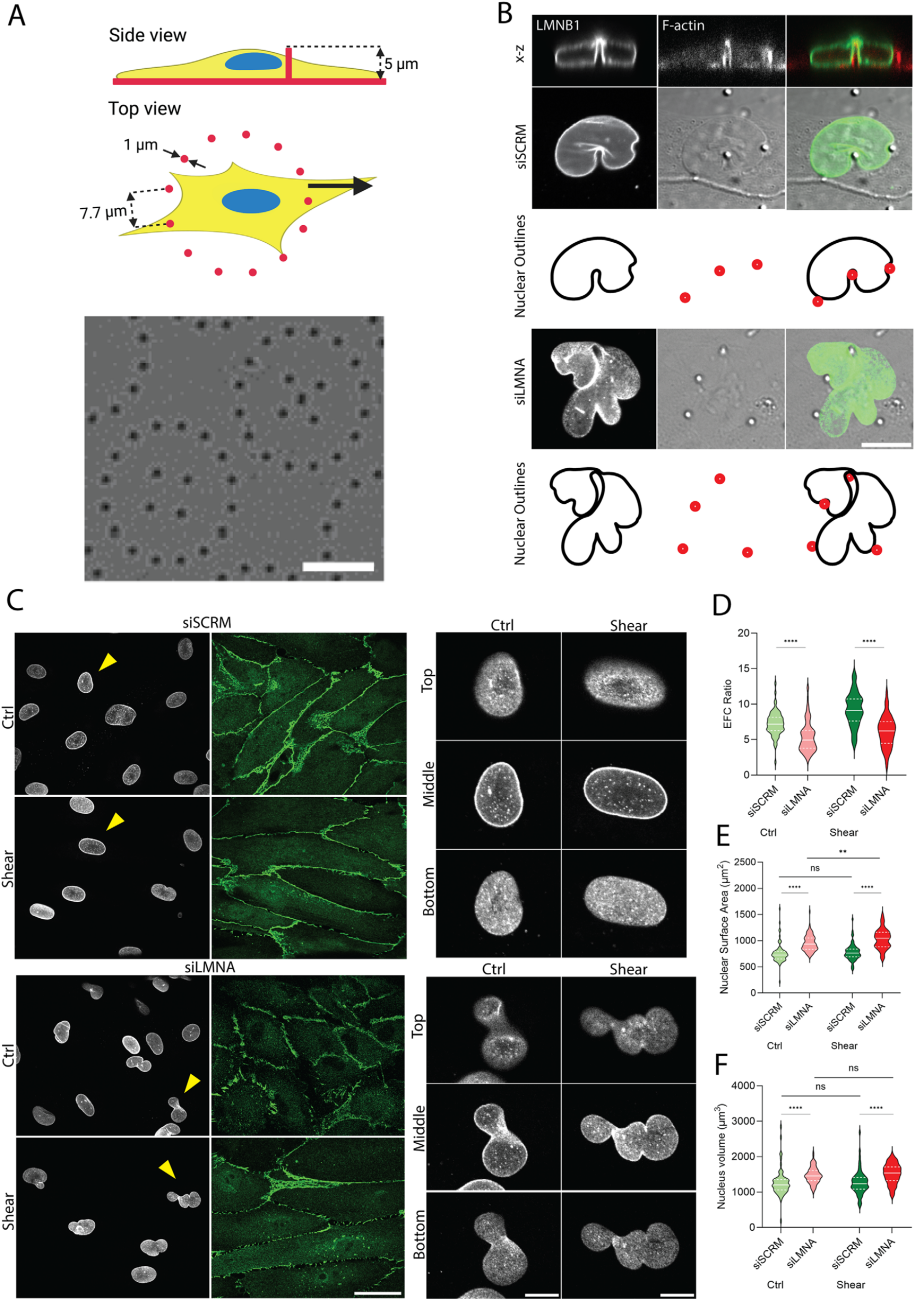
Lamin A/C confers surface tension on the nuclear lamina. (A) Schematic and brightfield images of a circular pattern of 5‐µm‐tall PDMS microposts. Scale bar on the brightfield image is 20 µm. (B) Confocal images showing stained lamin B1 in siRNA‐transfected HUVECs (siSCRM and siLMNA) merged with a confocal image of F‐actin and DIC image. The scale bar is 10 µm. Nuclear outlines show the indentations of the nuclei and positions of the microposts. (C) Confocal images of lamin B1 (gray) and VE‐Cadherin (green) in siRNA‐transfected HUVECs (siSCRM and siLMNA) in two different conditions, control and shear stress. The scale bar is 40 µm. The arrows indicate the nuclei shown in the closeup views (scale bar in the closeup view is 10 µm). Violin plots show (D) nuclear EFC ratio, (E) surface area, and (F) volume for siRNA‐transfected HUVECs (siSCRM and siLMNA) cultured in control and shear stress conditions corresponding to (D). *n* = 69, 103, 65, 54 from three independent experiments. ns *p* > 0.05, ^**^ *p* < 0.01, ^****^ *p* < 0.0001 by Mann‐Whitney U test.

To test whether lamin A/C confers surface tension on the nucleus in ECs as we have found in cancer cells and fibroblasts [11, 15], we cultured cells on closely spaced PDMS microposts [15] that pose obstacles to cells, and in particular to the large nucleus, as the cells spread or migrate. A schematic and brightfield image of the setup shows PDMS microposts of ∼5 µm height patterned in circular arrays (Figure 2A). In control HUVECs, the nuclear lamina indented by microposts displayed a smooth, drop‐like contour with free surfaces of approximately constant curvature, consistent with the presence of in‐plane surface tension balanced by a nuclear pressure (Figure 2B and outlines, top rows) [11, 19, 20]. In contrast, siLMNA‐transfected cells exhibited irregular laminar contours and retained deep indentations even in regions not contacting microposts (Figure 2B and outlines, bottom rows), suggesting poor shape recovery post de‐indentation, and a reduction in surface tension (siRNA transfection was optimized to reduce levels of lamin A/C in HUVECs by about 60% while avoiding toxicity to the primary HUVECs, Figure S2A). These findings are consistent with the drop model, in which lamin A/C confers surface tension on the nucleus.

We next examined the effect of lamin A/C depletion on the shapes of lamin B1‐stained nuclei in endothelial monolayers under shear stress. Control nuclei transfected with scrambled siRNA were generally circular with a smooth lamina in the absence of shear stress (Figure 2C, top rows). The application of shear stress elongated cells and nuclei while maintaining their smooth lamina. In contrast, the nuclear lamina in lamin A/C depleted cells was irregular in shape, both in the absence of shear and under shear (Figure 2C, bottom rows). Expression of GFP lamin A in lamin A/C depleted cells rescued the abnormal nuclear shapes (Figure S3A,B). The quantification of nuclear EFC ratio revealed a population‐level decrease, supporting increased contour irregularity in lamin A/C depleted cells (Figure 2D). Interestingly, in both sheared and unsheared conditions, nuclear volume and surface area, both, were higher in lamin A/C depleted cells compared to control (Figure 2E,F). Further, while shear stress predictably reduced nuclear height in control cells (due to a decrease in the occurrence of wrinkled nuclei under shear), nuclear height in lamin A/C depleted cells under shear was lower compared to control (Figure S2B–D). The reduction in nuclear height in lamin A/C depleted cells is consistent with the prediction of the drop model that the lamin A/C‐containing inextensible lamina prevents further nuclear flattening once it is fully unfolded. Also, the area and volume measurements support the concept that lamin A/C is required for the constraints of constant area and volume during nuclear shape transformations in endothelial cells.

To test whether the results above were specific to A‐type lamins, we repeated the experiments with HUVECs depleted of lamin B1 and immunostained for lamin A/C. On PDMS microposts, nuclei in lamin B1‐depleted cells retained their smooth, drop‐like contours similar to controls (Figure S4A,B), indicating that loss of lamin B1 does not impair the lamina's ability to sustain surface tension. Applying shear stress reduced the occurrence of wrinkled nuclei in siLMNB1‐transfected cells as well as controls (Figure S4C). Quantification confirmed that the nuclear EFC ratio increased upon shear application in siLMNB1‐transfected cells (Figure S4D). These findings demonstrate that A‐type lamins, and not lamin B1, are required for laminar surface tension.

### 2.3 Predictions of HUVEC Nuclear Shapes Using the Drop Model

**FIGURE 3:**
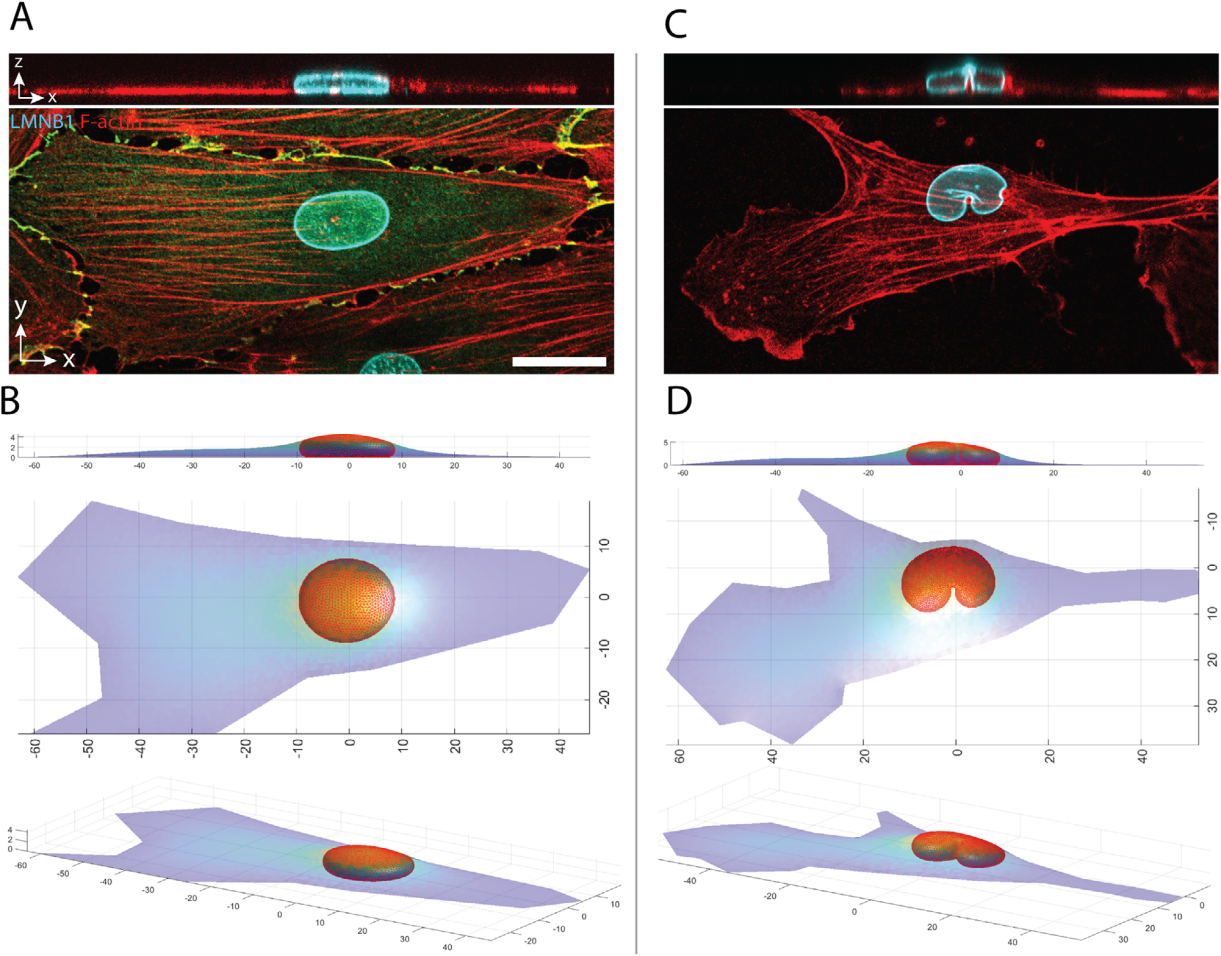
Model‐based calculation of nuclear and cell geometry based on the drop model. (A) Confocal images of lamin B1 (cyan) and F‐actin (red) in HUVEC cultured in the cell monolayer. Scale bar is 20 µm. (B) Calculated cell and nuclear shape compared to experimentally observed shape in (A). (C) Confocal images of lamin B1 (cyan) and F‐actin (red) in a HUVEC cell with the nucleus indented by a one micron‐diameter micropost. (D) Calculated cell and nuclear shapes are compared to experimentally observed shapes in (C). Consistent with the assumptions of the nuclear drop model in confinement under the cell cortex, the calculated cell and nuclear shapes were determined by minimizing the surface area of the triangulated cell surface (see Materials and Methods) under the constraints of constant cell volume (2800 mm^3^), nuclear volume (800 mm^3^), and nuclear surface area (1543 mm^2^, or 37% excess surface area over the area of a sphere of the same volume). The indented model nucleus (D) consisted of 1842 triangles, under a cell surface of 1299 triangles. The nucleus model in the monolayer cell (B) consisted of 1710 triangles, under a cell surface of 1691 triangles.

We hypothesized that the observed nuclear shapes in ECs are determined by the limiting shape established when all excess surface area of the nuclear lamina is smoothed while being confined under the tensed cell cortex of a fully spread cell. This limiting shape can be mathematically calculated by minimizing the surface area of the cell cortex for a given perimeter of the cell footprint, under the constraints of constant cell volume and laminar surface area [26]. The calculation was performed using an algorithm that minimizes the energy of the triangulated cell and nuclear surfaces. The algorithm prevents overlap between these surfaces and imposes a high energy penalty for deviations from the prescribed values of nuclear volume, cell volume, and nuclear surface area. The resulting flattened nuclear shapes closely resembled the observed ones, particularly in exhibiting a flattened apical nuclear surface in contact with the cell cortex, a highly curved cytoplasmic interface, and a flattened surface against the substratum (compare Figure 3A,B). These features are characteristic of a nucleus of fixed volume whose shape under vertical confinement is fully determined by the excess surface area of an inextensible nuclear lamina [26]. To further support the hypothesis that nuclear shapes in ECs are primarily determined by the inextensible lamina surface area, we also calculated the nuclear shape under lateral indentation by a one‐micron‐diameter micropillar. In this case as well, the computed nuclear shape (Figure 3D) closely resembled the experimentally observed shape (Figure 3C), exhibiting characteristics similar to those of a deformed liquid drop of fixed surface area [15].

### 2.4 Shear Stress Detaches Rounded Cells

**FIGURE 4:**
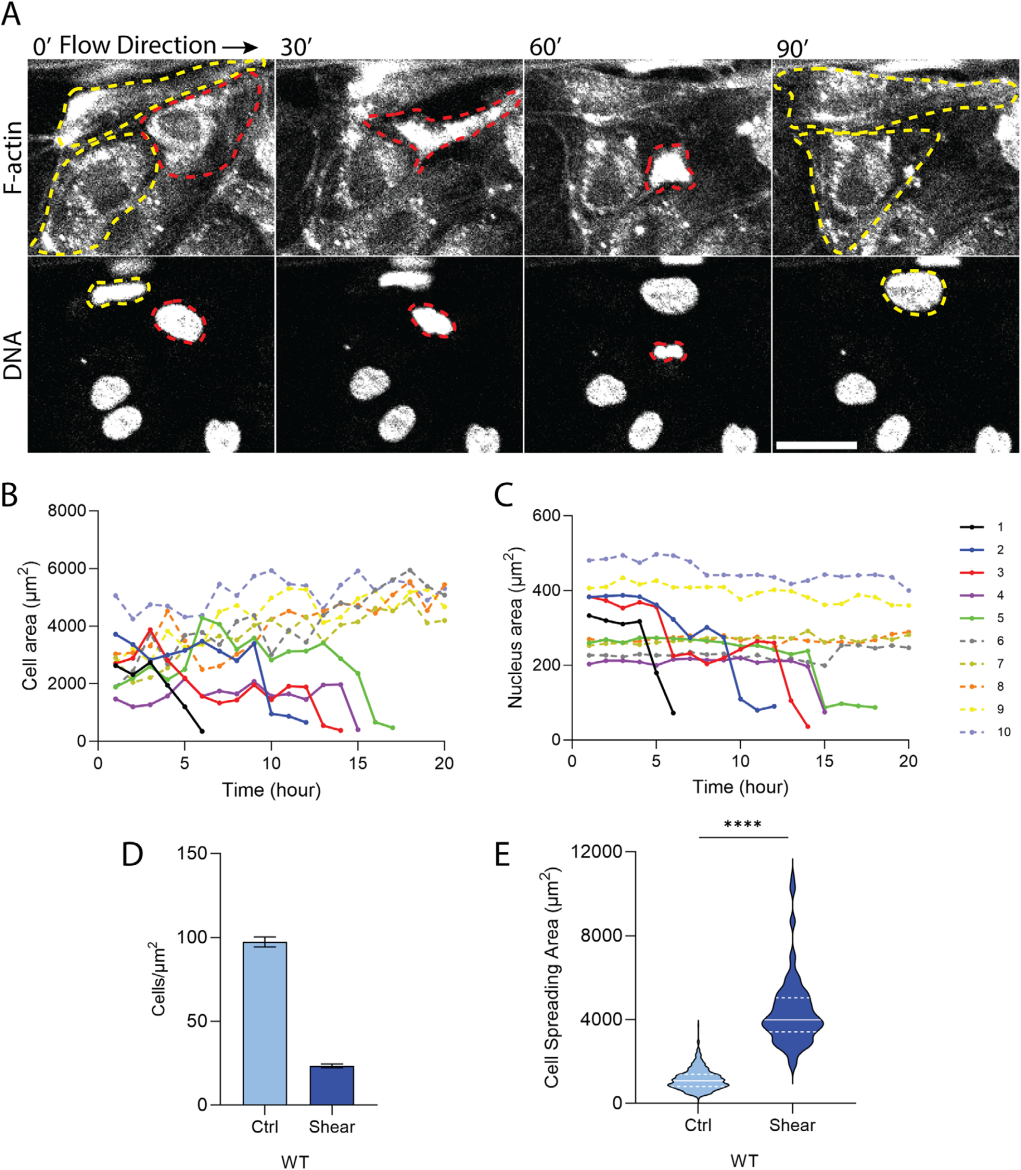
Shear stress causes cell detachment. (A) Time‐lapse confocal images of the HUVECs treated with SPY650‐FastAct and SPY595‐DNA in the monolayer under shear stress. Red dashed line outlines the cell and its nucleus that ultimately detaches. Yellow dashed lines indicate spread cells that remain behind. Scale bar is 40 µm. Quantification of (B) Cell area and (C) Nuclear area over time. Solid and dashed traces show detached and retained cells, respectively. (D) Quantification of the number of cells per area. *n* (analyzed images) = 8, 8 for WT (ctrl and shear) from three independent experiments. Error bars, SEM. (E) Violin plot showing the cell spreading area for ctrl and shear stress conditions. *n* = 461, 101 from three independent experiments. ^****^ *p* < 0.0001 by Mann–Whitney U test.

To determine whether shear stress directly compresses endothelial nuclei or instead reshapes the monolayer population, we analyzed nuclear morphology and cell occupancy under flow. Cell boundaries and nuclear morphology were visualized using SPY650‐FastAct and SPY595‐DNA (Cytoskeleton, Inc.), respectively, which are designed for live cell imaging [27]. Time‐lapse imaging (see Movie S1) revealed that cells which progressively rounded up upon application of shear flow eventually detached (Figure 4A top panel). The nuclei in these cells progressively reduced in their x‐y cross‐sectional area before detachment. As rounded cells have tall x‐z profiles (Figure 1), it is likely that shear stress detaches cells that pose a tall vertical cross‐section to the flow. These qualitative findings were confirmed by quantification of cell area over time: solid traces in Figure 4B are measurements from cells that detached, showing a drop in cell spreading area preceding disappearance, while dashed traces represent retained cells that fill in the gaps and cause a resultant increase in area. Corresponding decreases in nuclear cross‐section area were observed for the remaining cells, consistent with a vertical nuclear rounding preceding detachment (Figure 4C).

To validate these observations at the population level, we quantified cell density (cell numbers/area) in immunostained monolayers. Cell density decreased upon the application of shear, along with a corresponding increase in cell spreading area (Figure 4D,E). Together, these data demonstrate that shear stress does not compress already flattened nuclei, but rather selectively eliminates poorly spread cells. This results in a decrease in the mean nuclear height (as taller nuclei are eliminated from the population), a decrease in mean cell height, and enhanced cell spreading.

### 2.5 YAP Nuclear Localization Under Shear Reflects Enhanced Spreading of Retained Cells

**FIGURE 5:**
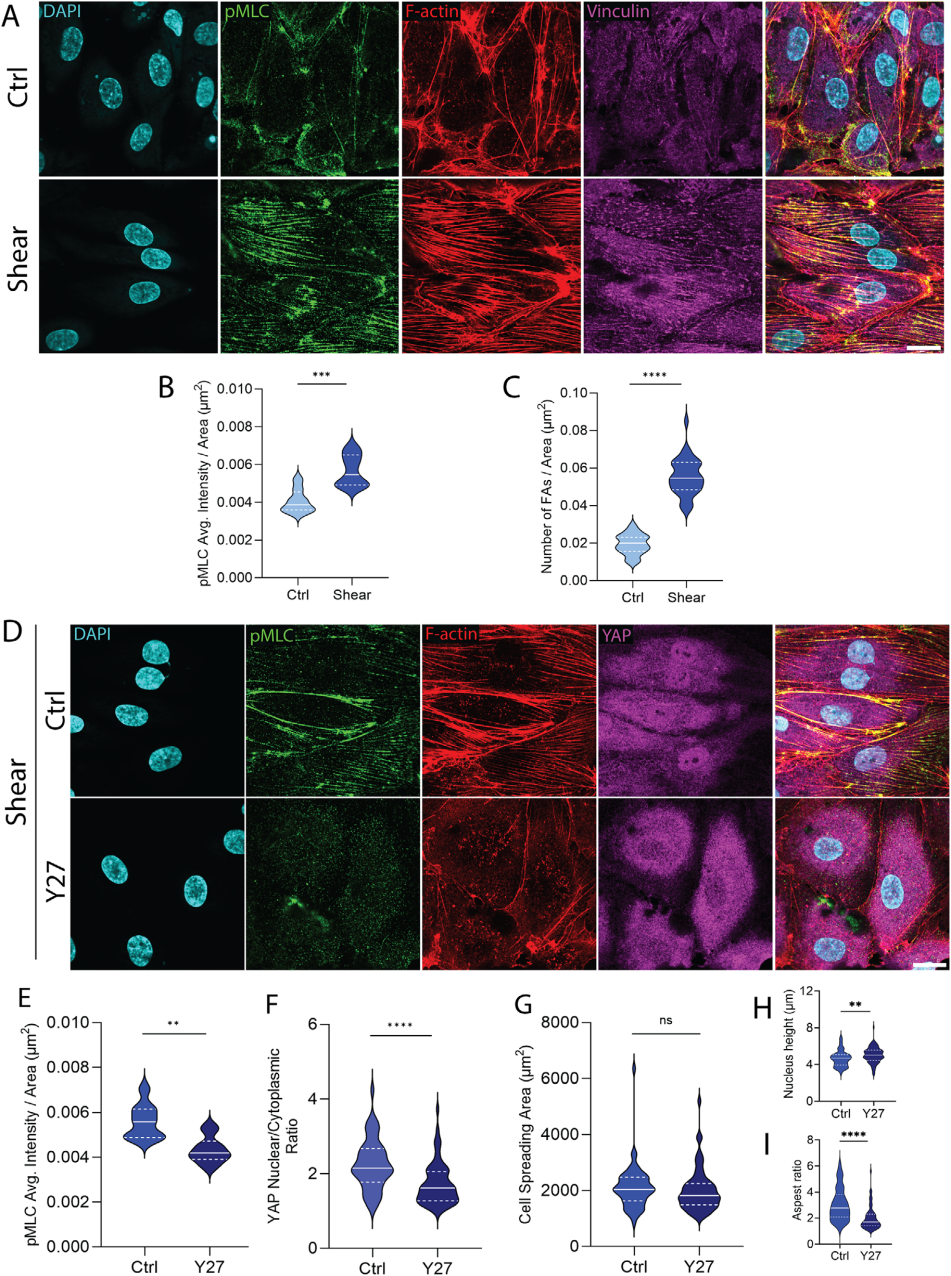
Shear stress enhances focal adhesion assembly and actomyosin contractility through ROCK‐dependent mechanisms. (A) Confocal images of stained DNA (cyan), pMLC (green), F‐actin (red), and FAs (magenta) in HUVECs monolayer; top: control, below: shear stress. The scale bar is 20 µm. Violin plots comparing (B) pMLC average intensity per unit area, and (C) number of FAs per unit area for HUVECs cultured in control (*n* = 33) and shear stress (*n* = 34) conditions from three independent experiments. ^***^ *p* < 0.001, ^****^ *p* < 0.0001 by Mann–Whitney U test. (D) Confocal images of stained DNA (cyan), pMLC (green), F‐actin (red), and YAP (magenta) in HUVEC monolayer under shear stress; top: control, bottom: 10 µM, Y27632 (Y27). The scale bar is 20 µm. Violin plots comparing (E) pMLC average intensity, (F) nuclear to cytoplasmic YAP intensity ratio, (G) cell spreading area, (H) nucleus height, and I) aspect ratio for HUVECs cultured under shear stress in two different conditions, control (*n* = 51) and Y27 (*n* = 60) from three independent experiments. ns *p* > 0.05, ^**^ *p* < 0.01, ^****^ *p* < 0.0001 by Mann–Whitney U test.

Given that enhanced cell spreading is generally mediated by an increase in focal adhesion assembly and actomyosin tension [28, 29, 30], we performed experiments to quantify focal adhesion (FA) formation using vinculin immunostaining and actomyosin contractility by phospho‐myosin light chain (pMLC) immunolabeling in HUVEC monolayers subjected to shear stress. Shear stress caused a statistically significant increase in pMLC intensity normalized to cell area and in the number of FAs per cell area compared to static control (Figure 5A–C). Thus, shear stress enhances integrin–ECM engagement and cytoskeletal tension, consistent with the observed increase in HUVEC spreading under flow.

As ROCK‐mediated contractility is known to mediate mechanosensitive YAP activation [17, 31, 32], we further tested whether ROCK‐mediated contractility is required for shear‐induced effects on YAP nuclear translocation. HUVEC monolayers were exposed to shear for 20 h (1 dyne/cm^2^) and treated with the ROCK inhibitor Y‐27632 (10 µM) during the final hour of flow. ROCK inhibition significantly reduced pMLC intensity and suppressed the nuclear‐to‐cytoplasmic YAP ratio (Figure 5D–F). Cell spreading area was not significantly decreased upon Y27632 treatment, however, the cell aspect ratio reduced significantly, and nuclear height increased slightly (Figure 5G–I). These data suggest that shear‐induced detachment, which promotes an increase in contractility in the remaining spread cells, promotes YAP nuclear localization under shear.

**FIGURE 6:**
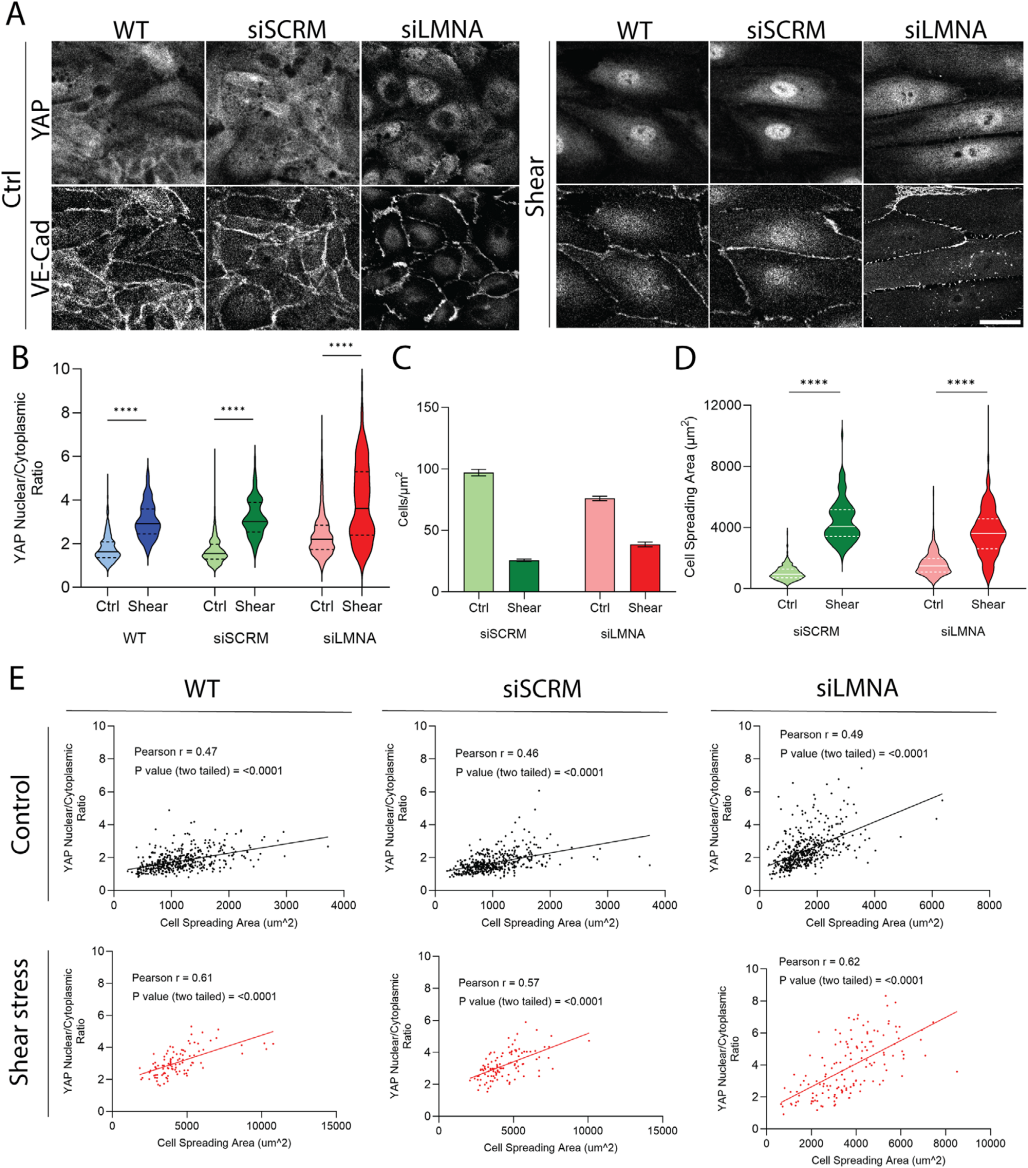
YAP activation under shear correlates with increased spreading area of the retained cells. (A) Confocal images of YAP and VE‐Cadherin for WT, siSCRM, and siLMNA groups in control and shear stress conditions. The scale bar is 30 µm. (B) The violin plot shows the nuclear to cytoplasmic YAP intensity ratio corresponding to the groups and conditions shown in (A). *n* = 461, 101, 457, 108, 486, 149 for WT (ctrl and shear), siSCRM (ctrl and shear), and siLMNA (ctrl and shear), respectively, from three independent experiments. ^****^ *p* < 0.0001 by Mann–Whitney U test. C) Quantification of the number of cells per area. *n* (analyzed images) = 7, 7, 10, 10 for siSCRM (ctrl and shear), and siLMNA (ctrl and shear), respectively. Error bars, SEM. D) Violin plots showing the cell spreading area for siSCRM, and siLMNA groups in control and shear stress conditions. *n* = 457, 108, 488, 149 for siSCRM (ctrl and shear) and siLMNA (ctrl and shear), respectively, from three independent experiments. ^****^ *p* < 0.0001 by Mann‐Whitney U test. E) Nuclear to cytoplasmic YAP intensity ratio, correlated to cell spreading area, is shown for WT, siSCRM, and siLMNA groups in control and shear stress conditions corresponding to (B). Pearson r values of linear fitting and p‐values for two‐tailed comparisons for the slopes are shown on the graphs.

Motivated by our recent findings that LMNA knockdown in cancer cells inhibits mechanosensitive localization of YAP to the nucleus [11], we asked whether lamin A/C depletion modulates YAP nuclear localization in HUVECs under shear stress. Application of shear stress increased the nuclear/cytoplasmic ratio of YAP significantly in wild‐type cells and in control cells transfected with scrambled siRNA. However, depletion of lamin A/C did not inhibit the shear‐induced increase in YAP levels in the nucleus (Figure 6A,B). Similar to WT cells (Figure 4D,E), applying shear stress reduced the density of cells transfected with siRNA (both siSCRM and siLMNA), with a corresponding increase in spreading area (Figure 6C,D). Consistent with this, a positive correlation between YAP nuclear/cytoplasmic ratio and cell spreading area was observed across each of the three conditions (Figure 6E). We confirmed that GFP‐lamin A transfection in lamin A‐depleted cells reduced the shear‐induced increase in YAP nuclear/cytoplasmic ratio relative to untransfected cells (Figure S31C). Additionally, lamin B1 depletion did not alter the shear‐induced YAP nuclear translocation nor increased cell spreading post shear (Figure S5A–C). These results show that neither lamin A/C nor lamin B1 is required for shear‐induced translocation of YAP to the endothelial nucleus. They again support the concept that YAP nuclear localization in ECs is mediated by an increase in spreading of the remaining cells under shear.

## 3 Discussion

Shear stress is a critical regulator of endothelial cell morphology, alignment, and signaling, all of which are essential for vascular homeostasis [1, 33]. While flow‐induced remodeling of the cytoskeleton is well established, the effect of the flow and shear stress on nuclear morphology has received less attention. We investigated the effects of shear stress on the HUVEC nucleus from the lens of the nuclear drop model. Consistent with the drop model, we found that the HUVEC nucleus possesses excess area that appears as wrinkles when its shape is rounded. These wrinkles disappear as the nucleus becomes flattened.

We also extend prior work on lamin A/C [34, 35, 36, 37, 38, 39, 40, 41, 42] by showing that in ECs, as in cancer cells [11], lamin A/C confers nuclear surface tension. On micropost arrays, nuclei from lamin A/C‐depleted cells showed persistent indentations and wrinkling. In contrast, the control (siSCRM) nucleus displayed drop‐like shapes characterized by free surfaces with nearly constant curvature, which is consistent with the presence of nuclear surface tension. Similar free nuclear surfaces of constant curvature were also observed under shear stress in the vessel‐on‐chip model. Thus, the drop model appears to be broadly applicable across cell types, and surface tension in the smooth nuclear lamina appears to be a conserved mechanical property.

Our findings support a central role for lamin A/C in maintaining nuclear shape integrity and mechanical behavior under flow shear stress. In siSCRM cells shown in Figure 2C, the nuclear lamina remained smooth and elliptical under both static and shear conditions, consistent with the presence of in‐plane surface tension that supports regular nuclear contours. However, lamin A/C depletion disrupted this tension, leading to pronounced laminar irregularities regardless of flow, as evidenced by a reduced EFC ratio and increased nuclear contour complexity (Figure S6).

Interestingly, the nuclei in lamin A/C depleted cells exhibited increased surface area and volume (Figure 2E,F), despite their reduced height (Figure S2B,D). This observation aligns with the predictions of the nuclear drop model: once the inextensible lamina has fully unfolded, further flattening is geometrically limited. In the absence of lamin A/C, the nucleus becomes more deformable, allowing surface area expansion and volume increase, while failing to maintain the mechanical constraints of constant area and volume. These results collectively suggest that lamin A/C is a key regulator of the mechanical constraints that govern endothelial nuclear deformation in response to cell spreading and shear.

Our simulation results suggest that nuclear shapes are geometrically (not mechanically) determined in the cell monolayer, similar to what we found previously for individual cells [10, 11, 43, 44]. Hence, cytoplasmic and nucleoplasmic mechanical properties are not primary determinants of nuclear shape. Our calculations show that nuclear shapes in ECs can be predicted by geometric constraints of fixed nuclear volume and lamina surface area under confinement by the cell cortex. These limiting shapes, characterized by a flattened apical surface and curved lateral and basal interfaces, align closely with experimentally observed morphologies. This agreement reinforces the idea that once the lamina becomes taut and unfolded, nuclear shape is no longer force‐dependent but geometrically determined. The ability of our model to also reproduce the invaginations formed by microposts further supports the idea that nuclei behave as pressurized drops when the lamina is fully unfolded. These findings are also consistent with our prior work showing that laminar unwrinkling, induced by cell spreading or matrix stiffness, results in the surface tension that shapes the nucleus [11, 20].

Our findings that shear stress selectively detaches vertically rounded, poorly spread endothelial cells from the monolayer align with the observations in epithelial systems, where cells undergo apical extrusion in response to crowding or mechanical imbalances [45, 46, 47]. Studies have shown that epithelial cells with reduced adhesion or increased mechanical stress are more likely to be eliminated from the monolayer through mechanically driven extrusion mechanisms [45, 48]. Such extrusion acts as a quality control process, maintaining tissue integrity by removing aberrant or overcrowded cells. Moreover, stress distributions around the topological defects can exacerbate the selection mechanisms. For example, Saw et al. [49] showed that mechanical competition within the epithelial monolayers leads to the extrusion of cells with higher contractility or impaired adhesion. Moreover, this process can be accelerated by external mechanical cues. Similarly, fluid shear stress in our vessel‐on‐chip system may enhance mechanical selection by shearing extruded HUVECs, promoting their detachment from the monolayer. This mechanical dropout would reduce the average nuclear height in the population, but not because individual nuclei are compressed. That is, nuclei that are maximally flattened as a result of their lamina becoming fully unfolded will not undergo further flattening owing to the high resistance of the nuclear lamina to extension and nuclear volume to compression [11]. This insight helps re‐interpret the findings by Barbee et al. who reported that shear stress reduces the average height of ECs and nuclei [3]. It is the loss of taller, wrinkled nuclei that causes the average height to fall.

We find that in static contexts, YAP is primarily in the cytoplasm. This result aligns with the observations of Das et al., in which YAP was excluded from the nucleus in confluent cultures of cells [50]. However, conflicting reports exist on the effect of shear stress on YAP translocation. Some studies show that laminar shear stress causes the entry of YAP into the nucleus [51, 52], while others have shown that laminar shear stress excludes YAP from the nucleus [53, 54, 55]. Still other studies have shown that disturbed flow causes YAP nuclear localization [54, 55, 56]. Our results suggest that in shear‐exposed HUVECs, YAP localization to the nucleus occurs regardless of lamin status and is instead associated with increased cell spreading among the retained population. Lamin A/C depletion does not inhibit YAP nuclear entry, but it indirectly promotes it by shifting the population toward more spread cells with increased focal adhesions and increased actomyosin tension, which are well‐known to promote YAP nuclear translocation [17, 32, 57, 58]. Consistent with this, Y27632 treatment inhibited the shear‐induced nuclear YAP localization. Additionally, the contrasting prior reports on the effects of shear stress on YAP nuclear localization in HUVECs may be reconcilable through measurements of shear‐induced cell detachment and associated cell spreading in the monolayer.

The finding that YAP translocates to the endothelial nucleus without necessitating nuclear deformation contrasts with previous studies that implicate force‐induced nuclear deformation in mediating YAP nuclear localization [59]. Shear‐induced cell spreading and the increased actomyosin tension may result in increased compressive stress from the actomyosin cortex on the nucleus, which results in an increased laminar tension [44]. This laminar tension could promote YAP nuclear localization through mechanisms that are currently unclear. Depletion of lamin A/C may result in altered tension in the nuclear membrane itself, thereby modulating pore properties and transport. Further, cell spreading may modulate the import and export rates of facilitated transport [60]. Alternatively, mechanisms that entirely operate in the cytoplasm, such as actomyosin‐mediated inhibition of the Hippo pathway, may account for the shear‐induced increase in YAP nuclear localization [61, 62]. For example, shear‐induced actomyosin fiber assembly, or altered F‐actin assembly in lamin A/C depleted cells [63], may reduce LATS1/2‐mediated phosphorylation of cytoplasmic YAP, thereby increasing cytoplasmic YAP availability and promote nuclear import.

Recent studies have suggested a potential role for apical stress fibers, or the F‐actin cap, in controlling nuclear morphology in HUVECs [64], building on prior studies by others [65, 66]. Shear stress can certainly alter the assembly of the F‐actin cap; however, it is unlikely that the F‐actin cap is responsible for the observed (minor) shear‐induced decrease in nuclear height. While models assume that the F‐actin cap compress the nucleus into flattened shapes, relaxation of compressive stress from stress fibers by laser ablation does not produce a relaxation of the compressed nuclear cross‐section [67]. Also, the flattening of the nucleus in the early stages of cell spreading is uncorrelated with apical and basal stress fibers, and nuclei can flatten in the absence of myosin activity [43]. Local protrusions cause proximal nuclear surfaces to move in the direction of the moving cell membrane, and the protruding nuclear surface relaxes back upon retraction of the protrusion [67]. These findings have led us to propose that moving boundaries of the cell transmit mechanical stress to the nuclear surface to deform it. Consistent with this, treatment with Y27632 caused a minor increase in nuclear height (although the nuclei remained flattened), but there was also a decrease in the elongation of the cell (Figure 5H,I). This inward movement of the cell boundaries along the long axis is predicted to transmit an inward stress to round up the nucleus [16, 43]. In any case, any such geometric effects are likely minor compared to the effect on the population mean of eliminating wrinkled nuclei. The reverse, however, is not true. That is, if shear were applied to a monolayer with already smooth flattened nuclei, additional spreading would not further flatten the nucleus owing to the constant‐volume and constant‐area constraints on the nucleus.

## 4 Methods

### 4.1 Endothelial Cell Culture

HUVECs were purchased from PromoCell (Cat.No. C‐12203), and they were used until passage 8. Endothelial growth media 2 (EGM2, PromoCell) was used for culturing HUVECs. For expanding the cells, the media was replaced every 48 h until the cells reached 80% confluency. HUVECs were subcultured and re‐plated at 12000–15000 cells/cm^2^ cell density and incubated in a 5% CO_2_ incubator at 37°C.

### 4.2 Cytochalasin D Treatment

Cytochalasin D treatment was performed on HUVECs to disrupt the actin cytoskeleton. HUVECs were trypsinized and seeded on the 35‐mm coverglass dishes (World Precision Instruments, LLC) coated with ECM mix (50 µg/mL fibronectin and 100 µg/mL type I rat tail collagen). Confluent dishes were incubated in a 4 µM solution of cytochalasin D in EGM2 media for 20 min. Because the cytochalasin D was dissolved in DMSO, the control cells were treated with the same amount of DMSO to prevent its effect on the experiment. The control and treated groups were fixed by 4% paraformaldehyde for further immunostaining and imaging process.

### 4.3 Micropost Fabrication

To fabricate the micropost stamps, a mixture of Sylgard 184 Silicone Elastomer (Dow Corning) with its Curing Agent at a 10:1 ratio was degassed and poured on the micropost mold to be cured at 65°C overnight [15]. Once cured, the micropost stamps were peeled off and used for upright microposts fabrication on 35‐mm coverglass dishes (World Precision Instruments, LLC). The dishes were coated with ECM mix (50 µg/mL fibronectin and 100 µg/mL type I rat tail collagen) and incubated at 37°C in a 5% CO_2_ incubator for an hour, washed thrice with 1X PBS, and then the HUVECs were passaged and incubated overnight at 37°C. Then, the cells were fixed for immunofluorescence process and microscopy.

### 4.4 Vessel‐Chip Fabrication and Applying Shear Stress to the HUVECs

Soft lithography of polydimethylsiloxane (Dow Corning) was used to fabricate microfluidic channels (200 µm width, 200 µm height, and 20 mm length) [68]. The fabricated polydimethylsiloxane slabs containing the channel patterns were cut out, and the inlet and outlet holes were punched using a 1 mm hole puncher (Miltex Instruments). The slabs were then bonded to the cover glasses (Corning), to be able to do imaging by 60X, coated with polydimethylsiloxane using a 100‐W plasma cleaner (Thierry Zepto, Diener Electronic). The devices were filled with the ECM mix (50 µg/mL fibronectin and 100 µg/mL type I rat tail collagen) and incubated at 37°C in a 5% CO_2_ incubator for an hour prior to washing out the fibronectin‐collagen solution with 1X PBS. HUVECs were detached in the confluent flasks and were seeded on the microfluidic channels at a concentration of 8–10 million cells/mL. The cells were attached to the basal matrix coating of channels by incubating the devices for an hour at 37°C in a 5% CO_2_ incubator. The same process was performed with a new flask of cells for an hour while the devices are kept upside‐down to attach the cells on all the surfaces of the channels. Homemade reservoirs by Falcon tubes were connected to the microfluidic channel inlets via metal PDMS couplers (DARWIN microfluidics) and PharMed BPT tubing (QOSINA, T2620). The outlets of cell‐seeded microfluidic channels were connected to a syringe pump (Harvard Apparatus, PHD Ultra) using the same types of metal PDMS couplers and BPT tubing and were perfused with the EGM2 media at ∼10 µL/min (wall shear stress τ:1 dyne/cm^2^), calculated based on Equation (1), where *Q* is the flow rate, *η* is fluid viscosity, and *w* and *h* are width and height of the channel, respectively. Elveflow and Fluigent microfluidic calculator tools were used to calculate the needed shear stress depending on the experiments.

(1) $$\tau =\frac{6Q}{wh^{2}}$$

Once the experiment was finished, cells were immediately fixed by 4% Paraformaldehyde for the following permeabilizing and immunostaining steps. The overall fabrication and flow setup are illustrated in Figure S7A–D (schematic).

### 4.5 ROCK Inhibition Under Shear Stress

Confluent HUVEC monolayers were subjected to steady laminar shear stress (∼1 dyne.cm^−^^2^) for a total of 20 h, as described above. During the final hour of perfusion, the medium was supplemented with the ROCK inhibitor Y‐27632 (10 µM; MilliporeSigma, catalog SCM075) without interrupting perfusion. At the end of the experiment, cells were fixed with 4% paraformaldehyde and immunostained for YAP, pMLC, and F‐actin.

### 4.6 Immunofluorescence and Microscopy

The cells cultured on 35‐mm cover glass dishes and microfluidic channels were fixed by 4% paraformaldehyde (Alfa Aesar) for 15 min at room temperature. A permeabilization buffer containing 0.1% Triton X‐100 (ThermoFisher Scientific) in 1X PBS was kept in the dishes and the channels for 30 min to permeabilize the cells before blocking for 1 h at room temperature (SuperBlock Blocking Buffer, Thermo Scientific). HUVECs were incubated with the following primary antibodies overnight at 4°C; rabbit anti‐lamin B1 (abcam, ab16048; 1:1000), rabbit anti‐lamin A/C (abcam, ab108595; 1:1000), mouse anti‐YAP (Santa Cruz Biotechnology, sc‐101199; 1:100), rabbit anti‐Phospho‐Myosin Light Chain 2 (Ser19) (Cell Signaling Technology, #3671; 1:200), mouse anti‐vinculin (Santa Cruz Biotechnology, sc‐73614; 1:200). Then, the cells were washed with PBS thrice and kept in the following secondary antibodies for 2 h at room temperature; Alexa Fluor 405 goat anti‐rabbit (Invitrogen, A48254; dilution 1:200), Alexa Fluor 647 goat antimouse (Invitrogen, A21235; dilution 1:200), Alexa Fluor 488 goat anti‐rabbit (Thermo Fisher Scientific, A11034; dilution 1:200). VE‐Cadherin was stained with Human VE‐Cadherin Alexa Fluor 488‐conjugated Antibody (R&D Systems, FAB9381G; 1:200) overnight at 4°C. F‐actin was stained with Alexa Fluor 594 Phalloidin (Invitrogen, A12381; dilution 1:200) for 30 min at room temperature. The stained cells on coverglass dishes and microfluidic channels were imaged by an Olympus FV3000 confocal microscope using 20×/0.8 and 60×/1.5 NA oil‐immersion objectives. Moreover, a z‐step size of 130 nm was used for 3D confocal imaging to satisfy the Nyquist criterion.

### 4.7 LMNA and LMNB1 Gene Knockdown Using siRNA Transfection

To knockdown gene in HUVECs, cells were cultured in an antibiotic‐free EGM2, and grown to ∼80% confluency. To minimize cytotoxicity and cell death, we tested multiple transfection reagents and different concentrations of siRNA. Transfections were performed in reduced‐serum Opti‐MEM (Gibco) using DharmaFECT #4 (Dharmacon Reagents, T‐2001‐01). Cells were transfected with 0.5% siRNA concentration (siGENOME control pool Non‐targeting #2 (Dharmacon Reagents, D‐001206‐14‐05), target sequences: UAAGGCUAUGAAGAGAUAC, AUGUAUUGGCCUGUAUUAG, AUGAACGUGAAUUGCUCAA, UGGUUUACAUGUCGACUAA; 0.5% concentration of siGENOME SMARTpool Human LMNA (Dharmacon Reagents, M‐004978‐01‐0005), target sequences: GAAGGAGGGUGACCUGAUA; 2% concentration of siGENOME SMARTpool Human LMNB1 (Dharmacon Reagents, D‐005270‐01), target sequences: GAAGGAAUCUGAUCUUAAU. After the replacement of culture medium with transfection solution, the cells were incubated in a 5% CO_2_ incubator at 37°C for 3 days. After 72 h, the cells were detached for the purpose of conducting a polymerase chain reaction (PCR) assay to validate siRNA knockdown and were seeded on the microposts or microfluidic channels to perform the related experiments.

### 4.8 Reverse Transcriptase‐Quantitative PCR

To verify the siRNA knockdown efficiency, the cells were trypsinized and lysed by a RNeasy Plus Mini Kit (Qiagen), followed by RNA purification and quantification (NanoDrop One^C^, Thermo Scientific). The preparation of cDNA was performed by mixing RNA with reverse transcriptase, oligo primers, and nucleotide triphosphates (dNTPs) (iScript Adv cDNA Kit, cat. #1725038, Bio‐Rad) in nuclease‐free water (PCR‐grade, Invitrogen). The resulted reaction mixture was incubated in a thermocycler (C1000 Touch, Bio‐Rad) at the proper duration and temperature for reverse transcription. Following forward and reverse primer sets were used to target the genes of interest cDNA: (GAPD, forward (5′–GAGTCAACGGATTTGGTCGT–3′), reverse (5′– TTGATTTTGGAGGGATCTCG –3′), (RealTimePrimers, VHPS‐3541); LMNA FW: ATGAGGACCAGGTGGAGCAGTA, RS: ACCAGGTTGCTGTTCCTC‐TCAG (Ref# 460260112 & 13, IDT); LMNB1 FW: GAGAGCAACATGATGCCCAAGTG, RV: GTTCTTCCCTGGCACTGTTG (Ref# 460260114 & 15, IDT)). The PCR reaction combined cDNA, primers, DNA polymerase, dNTPs, and iQ SYBR Green Supermix reaction buffer (cat. #1708880, Bio‐Rad). PCR amplification followed the protocol provided by Bio‐Rad. Gene expression levels for the genes of interest were quantified using standard curves and Ct values. These levels were then normalized to the expression level of GAPDH within the treatment group, relative to the normalized gene expression levels in the siSCRM group.

### 4.9 GFP‐Lamin A Rescue Assay

The pBABE‐puro‐GFP‐wt‐lamin A plasmid was purchased from Addgene (plasmid #17662). GFP lamin A was expressed in lamin A/C depleted HUVEC cells by retroviral transduction. Phoenix‐Ampho cells were transfected with pBABE‐puro‐GFP‐wt‐lamin A plasmid to produce viral particles using Lipofectamine 3000 (Thermo Fisher Scientific, Waltham, MA). Transfected Phoenix Ampho cells were incubated at 37°C for 8 h. Transfection medium was replaced with fresh culture medium, and cells were incubated at 32°C for 48 h. Culture medium containing packaged viral particles was collected and filtered using a 0.45‐µm filter (Thermo Fisher Scientific, Waltham, MA). Filtered medium was supplemented with polybrene (2.5 µg/mL; Sigma‐Aldrich, Saint Louis, MO) and added to the lamin A/C depleted HUVEC cells. The target cells were incubated 37°C for 48 h. The infection medium was replaced with fresh culture medium, and cells were maintained under standard culture conditions.

### 4.10 Live Imaging Experiments on Vessel Chips and Detachment Analysis

Live‐cell imaging was conducted on vessel chips to monitor HUVEC nuclear and cell morphology, as well as cell detachment, under shear stress conditions. The vessel chip platform was positioned on the incubated stage of a confocal microscope, maintaining physiological conditions at 37°C with 5% CO_2_. All inlet and outlet connections were sealed using a two‐part epoxy adhesive (resin and hardener mixed at a 1:1 ratio) to ensure leak‐proof flow. A shear stress of 1 dyne/cm^2^ was applied using a syringe pump over a ∼24‐h period. Cell boundaries and nuclear morphology were visualized using SPY650‐FastAct and SPY595‐DNA (Cytoskeleton, Inc.), respectively, each diluted 1:1000 in growth medium. Time‐lapse imaging was performed by capturing images every 10 min over the 24‐h experiment, generating continuous live‐imaging files for downstream analysis.

### 4.11 Image Analysis

A customized MATLAB code was developed for morphometric and phenotypic analysis. Maximum‐projection images of the lamin channel were generated and segmented using an Otsu algorithm to create bulk nuclear masks for identifying nuclei. Nuclei that touched the image edges or overlapped with others were excluded from the analysis. To capture detailed features like folds and wrinkles on nuclear contours, a more precise segmentation approach was further employed that the intensity maximum on each normal line along the bulk nuclear periphery was traced as the lamina position, achieving sub‐pixel resolution for delineating precise nuclear contours. The resulting nuclear masks were applied to the lamin images to individually crop nuclei. To quantify nuclear height, mean lamin intensity was calculated for each z‐plane, and the onset and offset points from the background intensity were determined as the bottom and top confocal planes, respectively. Nuclear height was then calculated as the distance between these two confocal planes. Nuclear surface area and volume were quantified using MATLAB Image Processing Toolbox.

Nuclear irregularity was quantified using elliptical Fourier analysis [25], which approximates nuclear shapes by decomposing them into a series of harmonic ellipses [69]. The nuclear contour was fitted using a series of elliptic harmonics, defined by the Fourier series coefficients calculated from the x and y coordinates of the nuclear outline. A total of 15 harmonic ellipses were used, sufficient to accurately delineate even the most complex, irregular nuclei [25, 70]. The first‐frequency Fourier coefficients describe a rough ellipsoidal shape, and the Fourier coefficients at higher frequencies approximate more convoluted outlines. To quantify shape irregularity, the elliptic Fourier coefficient (EFC) ratio is calculated as the length sum of the major and minor semiaxes at the first frequency divided by the semiaxes length sum for the subsequent 14 harmonics at higher frequencies. In 3D z‐stack images, we accommodated the variation of nuclear irregularity across different z‐planes by extracting nuclear contours from the z‐plane at 75%, 50%, and 25% of nuclear height to calculate the EFC ratio at the top, middle, and bottom, respectively, and average the EFC ratios of these three planes for each nucleus.

Maximum‐projection images of the VE‐cadherin channel were generated and segmented using Cellpose [71] to create cell masks. Cell spreading area and aspect ratio were quantified using MATLAB Image Processing Toolbox. To quantify the nuclear to cytoplasmic ratio of YAP, the nuclear and cell masks were applied to the maximum‐projection images of the YAP channel, and the nuclear to cytoplasmic YAP ratio was calculated as: [(Nuclear intensity)−(Background intensity)] ÷ [(Cytoplasmic intensity)−(Background intensity)].

To generate the color‐coded plots of the endothelial monolayer under control and shear stress conditions, 3D confocal image stacks were collected. These images included four channels: Lamin B1, VE_Cadherin, F‐actin, and YAP. These four channels were combined in each 3‐D image. Among various methods tested in MATLAB, the “imbinarize” function yielded the most consistent segmentation across variable cell shapes. To verify segmentation results, we created an image with a trace of the segmentation over the original image at an x‐z slice. Here, we ensured that the segmentation did not cut off any cell in the process. To avoid edge artifacts, peripheral regions adjacent to channel walls were excluded using a predefined cropping boundary. Height maps were generated by calculating the vertical distance between the apical and basal cell surfaces at each pixel and coloring by the height in each area of the image. Regions without cell coverage were interpolated using the mean height of adjacent pixels. Spatial resolution was down‐sampled to suppress high‐frequency noise. For each condition, the standard deviation of cell height was used as a metric of monolayer roughness, and color‐coded height maps were generated to visualize topographical variations.

Orientation analysis of F‐actin fibers and nuclear shape was performed using the F‐actin and lamin B1 fluorescence channels, respectively. Directionality was quantified using the Directionality plugin in ImageJ (NIH), which computes local orientation distributions based on the Fourier components of pixel intensity patterns.

ImageJ was used to measure the pMLC average intensity per unit area. Confocal z‐stack images of confluent HUVEC monolayers were collected at 60× magnification. After isolating the pMLC channel, the slices containing to the monolayer were averaged using the Z‐project (Average Intensity). Then, a mean gray value was measured over each region of interest (ROI). The average pMLC intensity per unit area was calculated by dividing the mean fluorescence intensity by the analyzed area. The same imaging and analysis parameters were used across all the samples.

ImageJ was used to measure the number of FAs per unit area. Fixed HUVECs were immunostained for vinculin and imaged at the basal plane (60× magnification). Rectangular ROI were selected within confluent areas. FAs were segmented from the vinculin channel by global thresholding (Otsu), followed by Analyze Particles with size 0.5–15 µm^2^ and circularity 0.1–1.00, excluding edge objects. The FA count (n) within the ROI was recorded and normalized by ROI area to yield FA density (FAs/µm^2^).

### 4.12 Modeling the Nuclear and Cell Shape Geometry

Nuclear shapes were calculated using the approach described in Dickinson and Lele [26], based on an algorithm of Pan et al. [72], which minimizes the surface area of a triangular Voronoi mesh *M*(*X*) (with *N* vertices at positions, $\;X={\left\{x_{i}\right\}}_{i=1}^{N}$) while simultaneously maintaining a centroidal Voronoi surface tessellation (with Voronoi cells, ${\left\{v_{i}\right\}}_{i=1}^{N}$). The vertex positions of the triangulated cell surface (*X_cell_*) and nuclear surface (*X_nuc_*) were simultaneously determined by minimizing the corresponding energies (*E_cell_* and *E_nuc_*, respectively), based on the following energy functional:

(2)

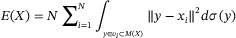

where the integrals are over the area of the Voronoi cells *v_i_* surrounding vertices, *x_i_*. The cell surface area was minimized while satisfying and nuclear volume and area constraints, *A_nuc_* (*X_nuc_*) = *A*_*nuc*,0_ , *V_cell_* (*X_cell_*) = *V*_*cell*,0_ , and *V_nuc_* (*X_nuc_*) = *V*_*nuc*,0_ , was achieved by minimizing the total energy function,

(3) $$\begin{array}{cc} E_{tot}\;\left(X_{cell},X_{nuc}\right) & =E_{cell}\;\left(X_{cell}\right)+\gamma {\left(\frac{A_{nuc}}{A_{nuc,0}}-1\right)}^{2}E_{nuc}\left(X_{nuc}\right)\\ & \;+\beta_{nuc}{\left(\frac{V_{nuc}}{V_{nuc,0}}-1\right)}^{2}+\beta_{cell}{\left(\frac{V_{cell}}{V_{cell,0}}-1\right)}^{2} \end{array}$$

Convergence towards the equilibrium cell and nuclear shapes that satisfied the area and volume constraints was achieved by progressively increasing the area and volume stiffness parameters, γ, β_*nuc*_, and β_*cell*_ until $|\frac{V_{nuc}}{V_{nuc,0}}-1|$, $|\frac{A_{nuc}}{A_{nuc,0}}-1|$, and $|\frac{V_{nuc}}{V_{nuc,0}}-1|$ were all less than 10^−3^. The cell and nuclear mesh surfaces were prevented from overlapping by a reflecting boundary algorithm described in Dickinson and Lele [26]. The boundary conditions at the cell adhesion perimeter (matched to the cell perimeter in the experimental image) were imposed by fixing vertex positions at triangulation edges, and the Voronoi surface tessellation was maintained during the optimization by flipping edges when two opposite angles of two adjacent triangles summed to be greater than p [72]. The initial mesh of nearly equilateral triangles was generated using the DISTMESH algorithm [73].

The primary parameter that determines nuclear shape for a given cell shape is the percent excess area (i.e., the surface area of the nucleus relative to that of a sphere of the same volume). For the calculations, we used the measured mean values of the nuclear volume and the surface area. The nuclear shape is weakly dependent on cell volume, and the cell volume was selected to provide a close fit to the measured x‐z profiles of the cell cortex.

For the case of the nucleus indented by the micropillar, the above algorithm was modified with the additional constraint preventing nuclear surface vertices from entering the pillar volume. The PDMS post was stepped slowly toward the final position at a rate slow enough to maintain near‐equilibrium at each step. During each iteration, any vertex crossing the micropost boundary was reflected to the nearest point on its surface.

### 4.13 Statistical Analysis

GraphPad Prism 9.5.0 was used to perform statistical analysis and plot the data. All the experiments had three replicates (*n* = 3) unless otherwise specified. The differences were considered significant for *p* < 0.05 and nonsignificant (NS) when *p* > 0.05. Any detailed information, conditions, and statistical tests are explained in the figure legends.

## Funding

This work was supported by the National Institutes of Health grant U01 CA225566 (to T.P.L. and R.B.D.), the Cancer Prevention and Research Institute of Texas Established investigator award RR200043 (to T.P.L.), the National Science Foundation awards 2412520 and 2226157 (to T.P.L.), and 2226156 to R.B.D, the US Army Medical Research (USAMRAA) Contract No. HT94252410432; NASA, BARDA, NIH, and USFDA, under Contract No. 80ARC023CA002; and NHLBI of NIH under Award Number R01HL157790 (to A.J.).

## Conflicts of Interest

The authors declare no conflicts of interest.

## Supporting information




**Supporting file 1**: smll72131‐sup‐0001‐SuppMat.docx.

**Supporting file 2**: smll72131‐sup‐0009‐FigureS1.tif.

**Supporting file 2**: smll72131‐sup‐0009‐FigureS2.tif.

**Supporting file 2**: smll72131‐sup‐0009‐FigureS3.tif.

**Supporting file 2**: smll72131‐sup‐0009‐FigureS4.tif.

**Supporting file 2**: smll72131‐sup‐0009‐FigureS5.tif.

**Supporting file 2**: smll72131‐sup‐0009‐MovieS6.avi.

**Supporting file 2**: smll72131‐sup‐0009‐FigureS7.png.

**Supporting file 2**: smll72131‐sup‐0009‐MovieS1.avi.

## Data Availability

The data that support the findings of this study are available from the corresponding author upon reasonable request.
