# Supplementary material for: Evidence for Drop‐Like Nuclear Deformation in Sheared Endothelial Monolayers
Source: Small. 2025 Dec 26;22(10):e06536. doi: 10.1002/smll.202506536 (PMC12910434; doi:10.1002/smll.202506536)
Supplement: Supplementary file 1 — Supporting file 1: smll72131‐sup‐0001‐SuppMat.docx. [file SMLL-22-e06536-s006.docx]

**Figure S1.** HUVECs orientation under shear stress. A) Graphs showing the orientation of F-actin fibers in three shear stress conditions – no shear (Ctrl), 0.1, and 1 dyne/cm^2^. Orientation was calculated by directionality tool in ImageJ. Each graph corresponds to the adjacent confocal image of F-actin. Scale bar is 50 µm. B) Graphs showing the orientation of cell nuclei in control (no shear) and shear stress conditions (1 dyne/cm^2^). The graphs correspond to the confocal images of lamin B1 stained nuclei. Scale bar is 30 µm.

**Figure S2.** A) The efficacy of lamin A/C knockdown by siRNA is shown for HUVECs. The relative expression of LMNA gene in siRNA-treated HUVECs to control HUVECs treated by scrambled siRNA (siSCRM) is calculated by normalizing of C_t_ values to the control gene expression, GAPDH, using the 2^-ΔΔCt^ method. The plot represents the mean from three biological replicates. Error bars, SEM. B) Three-dimensional view, generated by Imaris software, of the siRNA transfected (siSCRM and siLMNA) HUVEC nuclei in the monolayer (left) and the single view (right) of the nuclei (indicated by yellow arrow in the sheared monolayer). Single view of the 3D cross section and x-z view of the confocal image are shown. Scale bar is 10 µm. C) Violin plot shows the nuclear height in HUVECs cultured under static (ctrl) and shear stress conditions. *n* = 69, 65 for ctrl and shear, respectively, from three independent experiments. ****p < 0.0001 by Mann-Whitney U test. D) Violin plot shows the nucleus height in siSCRM (*n* = 65) and siLMNA (*n* = 48) conditions under shear stress, corresponding to the conditions shown in (B).

**Figure S3.** Rescue of Lamin A/C restores nuclear morphology and YAP mechanotransduction under shear stress. A) The efficacy of lamin A/C knockdown is shown for HUVECs. B) Collage of HUVEC nuclei taken by confocal microscope after LMNA knockdown (left) showing irregular nuclear morphologies, and of GFP–lamin A rescued cells (right) displaying restored smooth nuclear contours. Scale bar, 15 µm. C) Quantification of YAP nuclear/cytoplasmic ratio in shear-stressed siLMNA (KD) and GFP lamin A–rescued cells.

**Figure S4.** Lamin B1 knockdown does not affect nuclear morphology on the micropost device and under shear stress. A) The efficacy of lamin B1 knockdown by siRNA is shown for HUVECs. B) Confocal images showing stained lamin A/C in siRNA transfected HUVECs (siSCRM and siLMNB1) merged with DIC images on the microposts. The scale bar is 20 µm. C) Confocal images of lamin A/C (green) in siRNA transfected HUVECs (siSCRM and siLMNB1) in two different conditions, control and shear stress. The scale bar is 20 µm. D) Violin plot shows the comparison of nuclear EFC ratio for siRNA transfected HUVECs (siSCRM and siLMNB1) cultured in control and shear stress conditions corresponding to the groups shown in (C). n = 85, 91, 110, 73 for three independent experiments. ns p > 0.05, ****p < 0.0001 by Mann-Whitney U test.

**Figure S5.** Lamin B1 knockdown does not affect YAP translocation. A) Confocal images of the HUVECs stained for YAP in siRNA transfected cells (siSCRM and siLMNB1) in two different conditions, control and shear stress. The scale bar is 20 µm. Quantification of B) cell spreading area and C) YAP nuclear/cytoplasmic ratio, for siRNA transfected HUVECs (siSCRM and siLMNB1) cultured in control and shear stress conditions corresponding to the groups shown in (A). n = 74 ,71, 77, 57 from three independent experiments. ns p > 0.05, ****p < 0.0001 by Mann-Whitney U test.

**Figure S6.** Measurement of EFC ratio from confocal images of lamin B1-stained nuclei, corresponding to the conditions and quantifications shown in Figure 2 C and D. Two nuclei are shown for each of the conditions, from three confocal planes of top, middle, and bottom. The nuclear contours (shown in red) were calculated using a custom MATLAB code. The EFC ratio is calculated for each confocal plane and is shown as overlaid red text.

**Figure S7.** Schematic overview of the vessel-chip experimental workflow. A) A PDMS slab containing a pre-patterned microchannel is plasma-bonded to a PDMS-coated glass coverslip to form an enclosed channel. B) The inner side of the channel is functionalized with extracellular matrix (ECM) proteins to promote cell adhesion, followed by seeding of endothelial cells to establish a confluent monolayer mimicking the vascular endothelium. C) The microchannel seeded with cell is connected to a syringe pump and reservoir to enable continuous medium perfusion, generating defined levels of laminar shear stress that mimics the physiological flow conditions. D) After the desired flow exposure, cells are fixed and processed for immunofluorescence staining, followed by high resolution confocal microscopy for structural and molecular analyses.
