# Supplementary figures and images for: Evidence for Drop‐Like Nuclear Deformation in Sheared Endothelial Monolayers
Source: Small. 2025 Dec 26;22(10):e06536. doi: 10.1002/smll.202506536 (PMC12910434; doi:10.1002/smll.202506536)

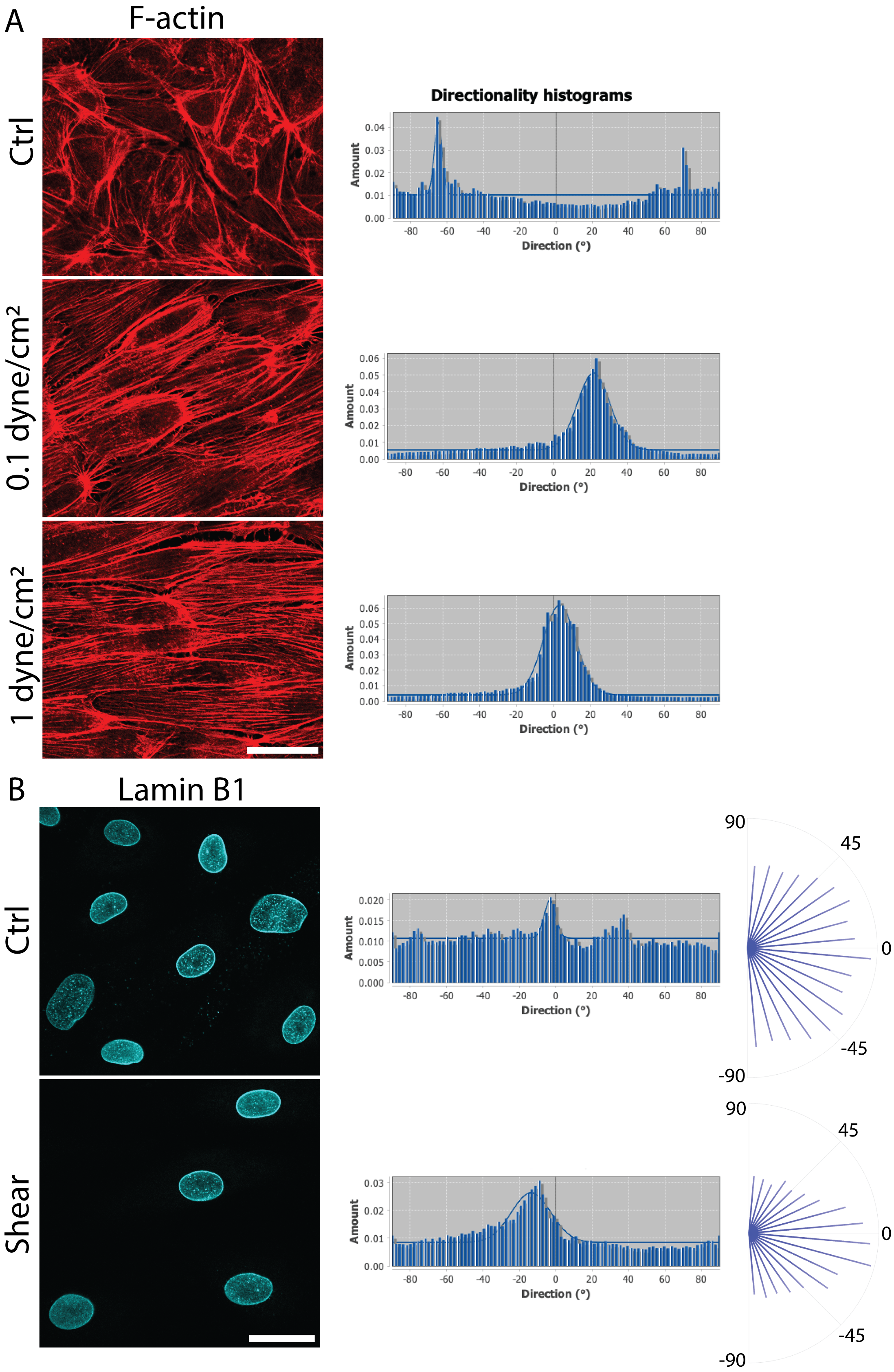

Supplement: Supplementary file 2 — Supporting file 2: smll72131‐sup‐0009‐FigureS1.tif. [file SMLL-22-e06536-s008.tif]

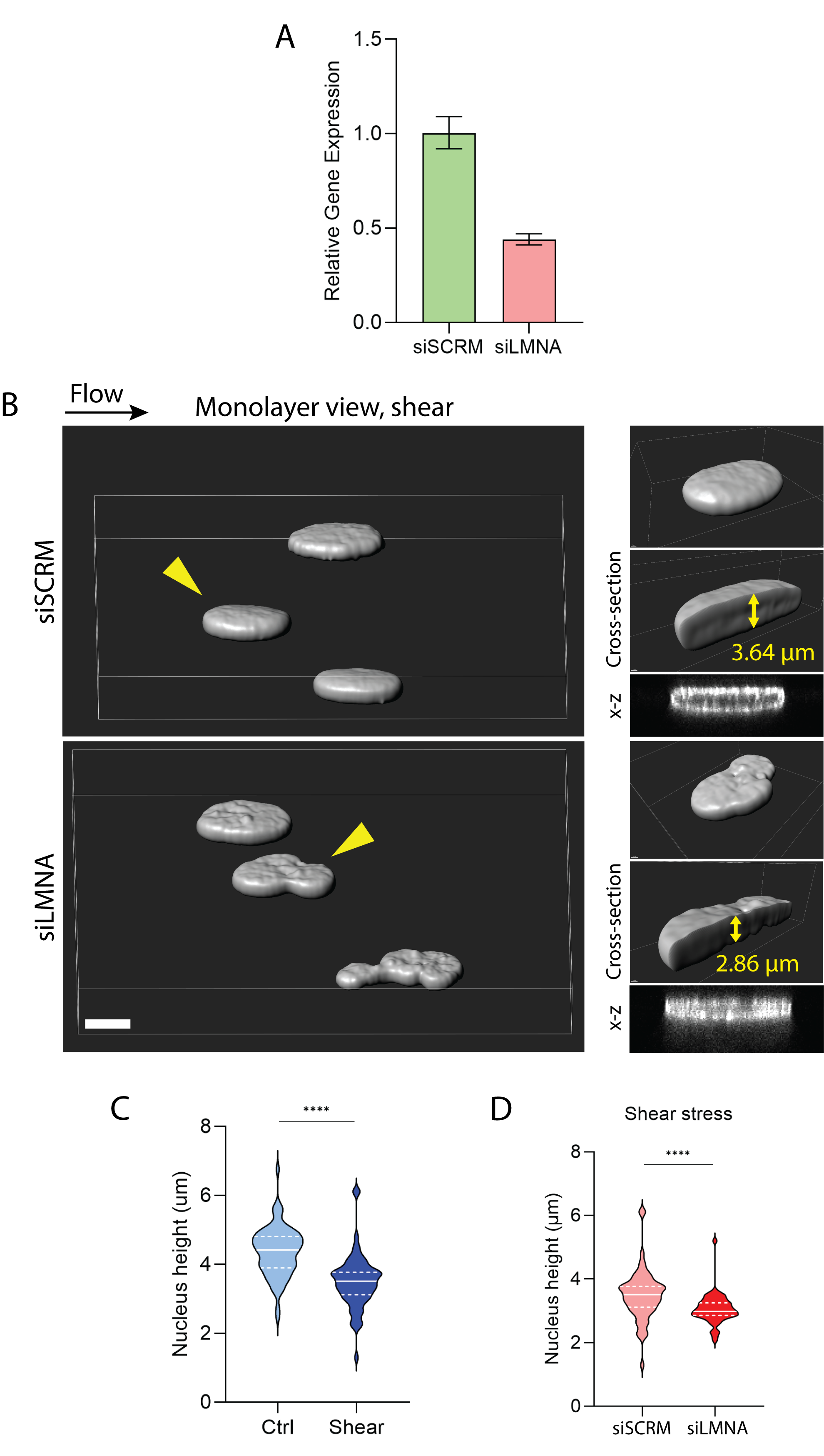

Supplement: Supplementary file 3 — Supporting file 2: smll72131‐sup‐0009‐FigureS2.tif. [file SMLL-22-e06536-s002.tif]

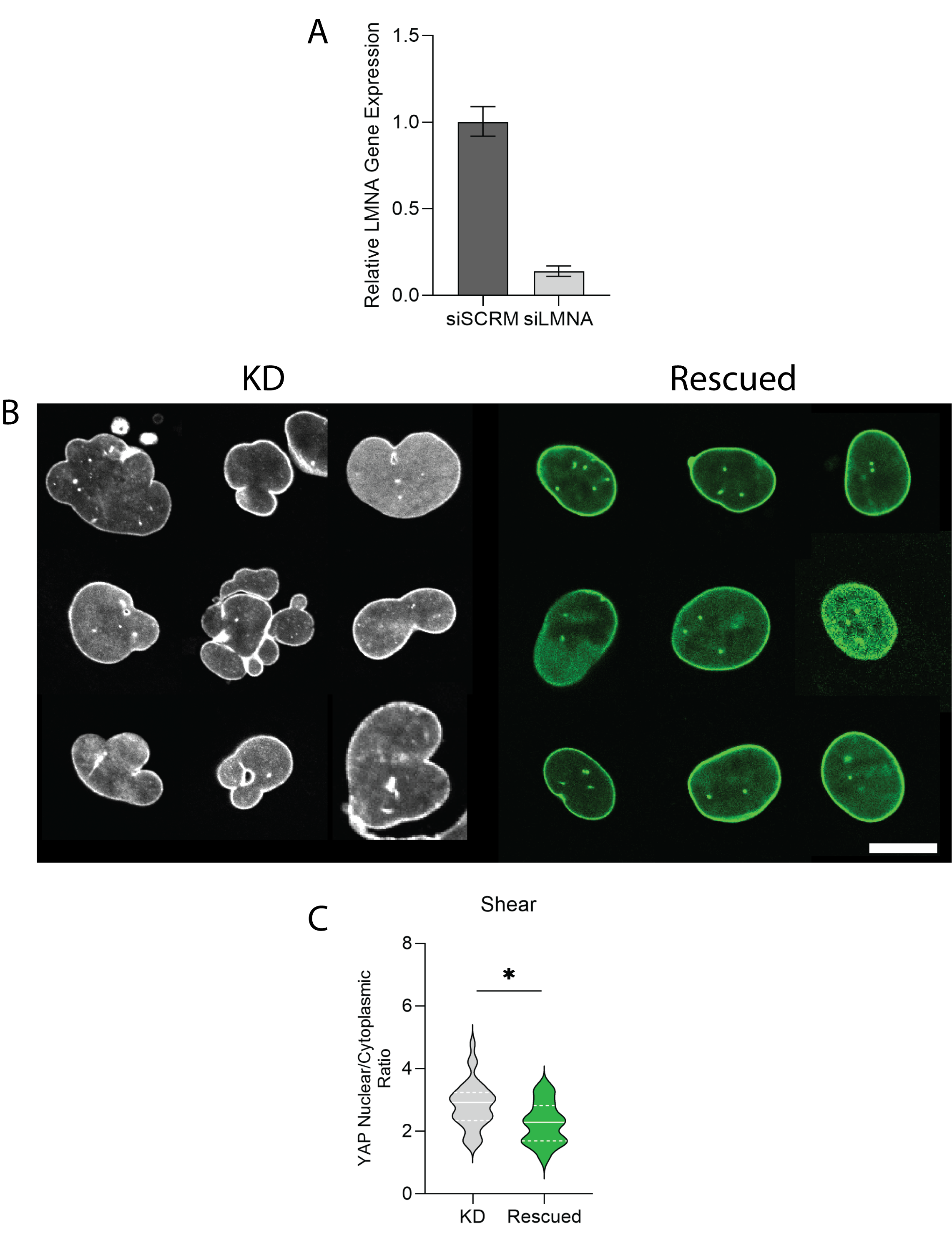

Supplement: Supplementary file 4 — Supporting file 2: smll72131‐sup‐0009‐FigureS3.tif. [file SMLL-22-e06536-s009.tif]

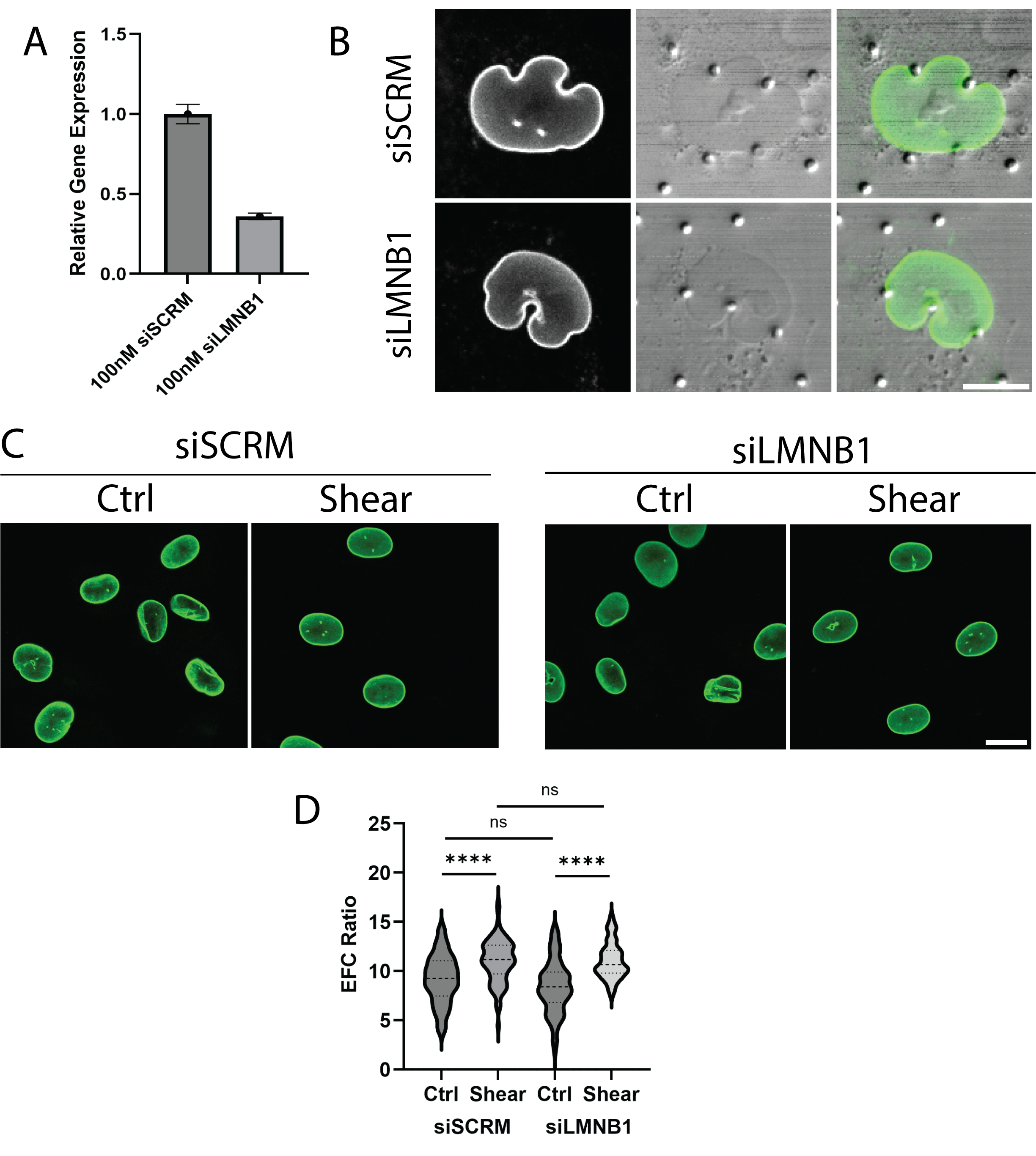

Supplement: Supplementary file 5 — Supporting file 2: smll72131‐sup‐0009‐FigureS4.tif. [file SMLL-22-e06536-s003.tif]

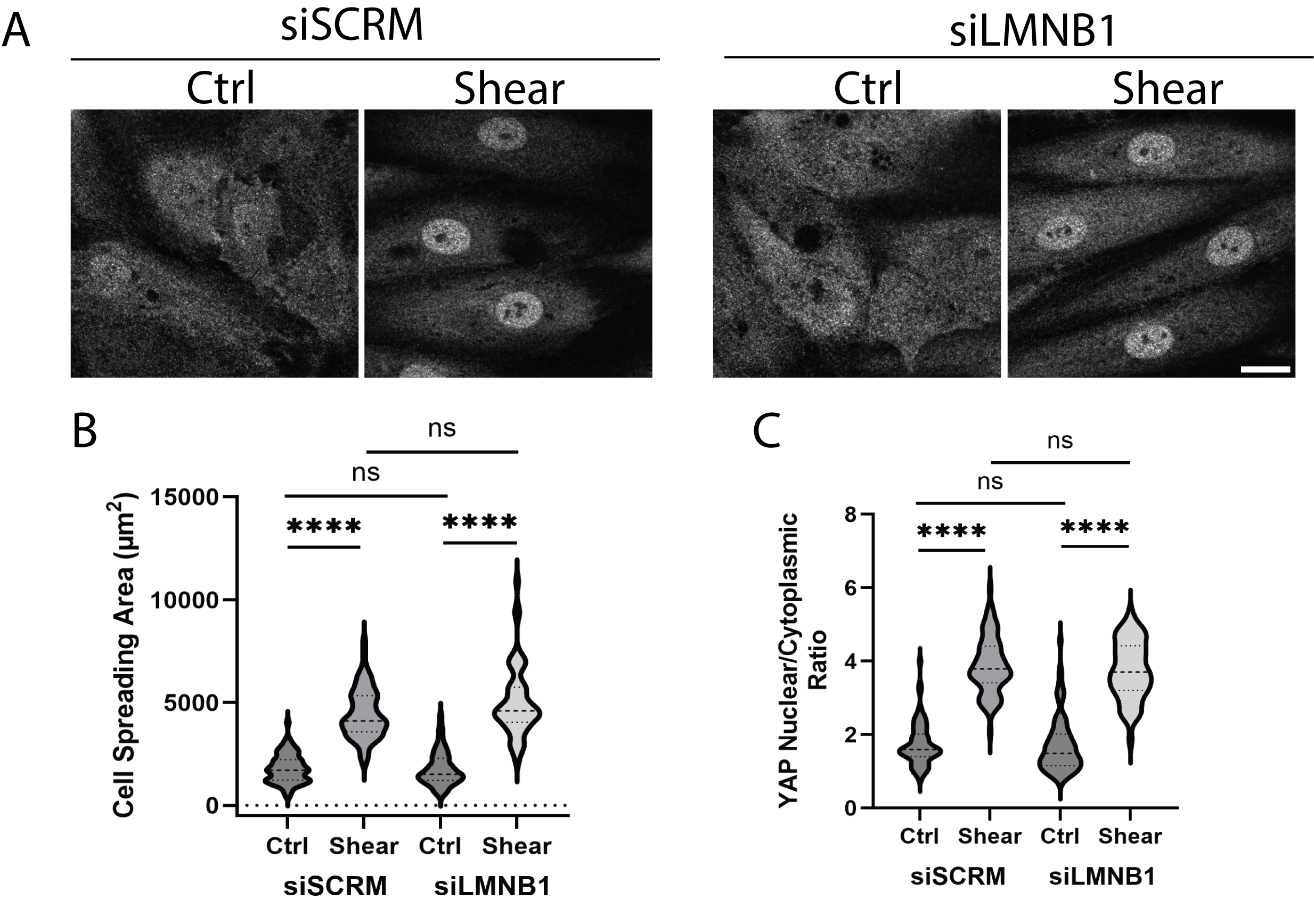

Supplement: Supplementary file 6 — Supporting file 2: smll72131‐sup‐0009‐FigureS5.tif. [file SMLL-22-e06536-s001.tif]

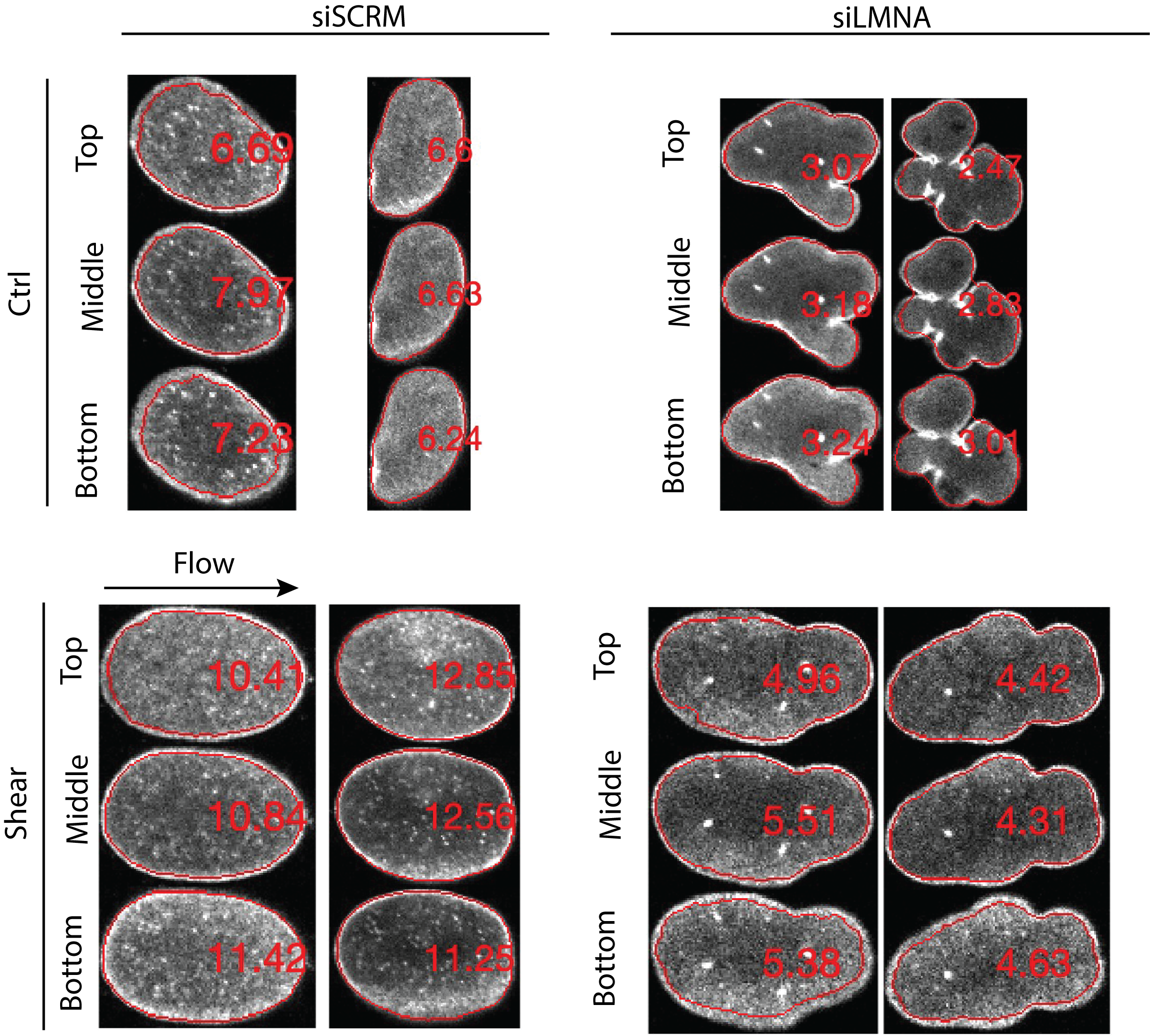

Supplement: Supplementary file 7 — Supporting file 2: smll72131‐sup‐0009‐MovieS6.avi. [file SMLL-22-e06536-s004.tif]

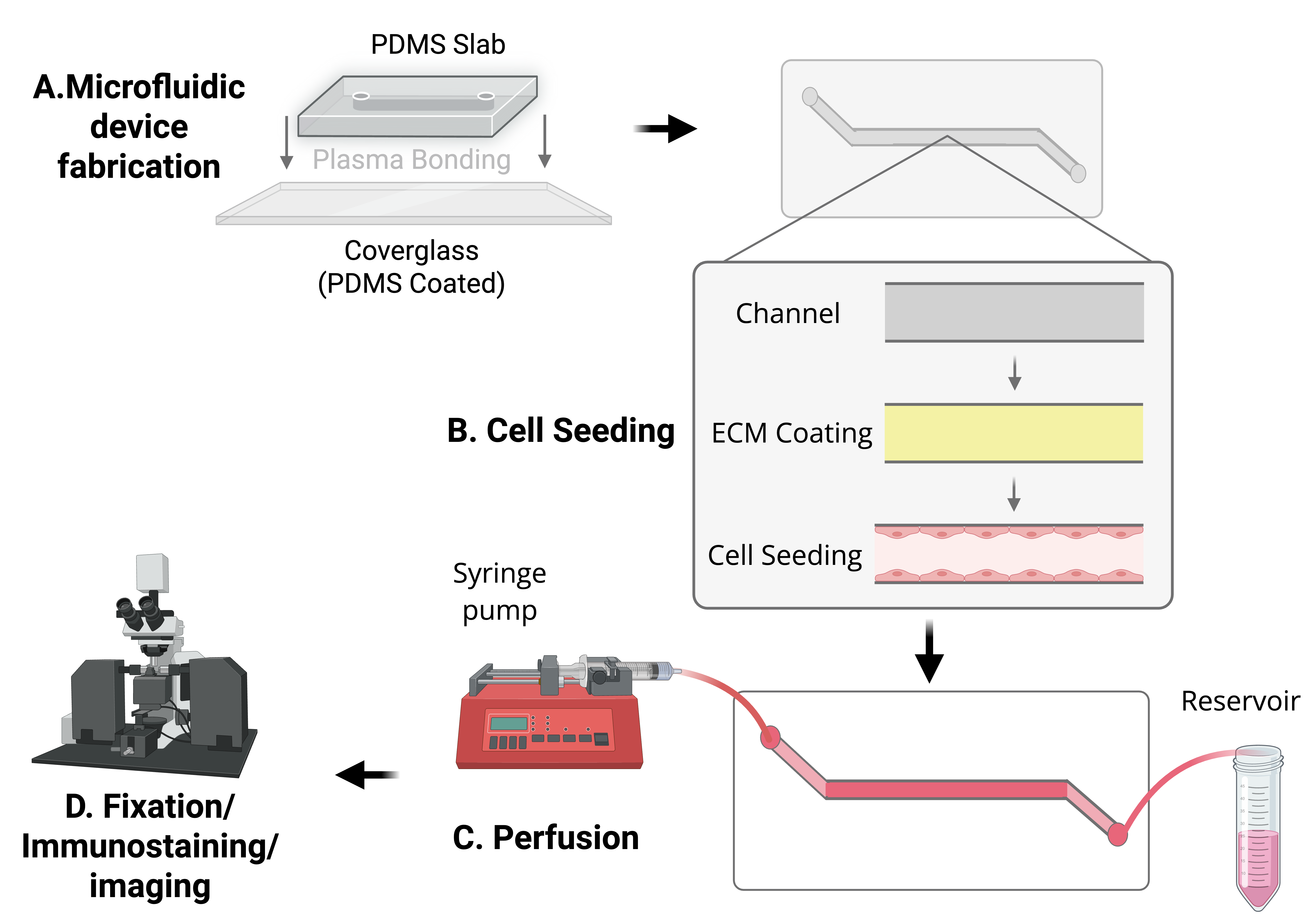

Supplement: Supplementary file 8 — Supporting file 2: smll72131‐sup‐0009‐FigureS7.png. [file SMLL-22-e06536-s007.png]
